# Hsp90 and associates shaping parasite biology

**DOI:** 10.1128/msphere.00329-25

**Published:** 2025-09-24

**Authors:** Abhilasha Gahlawat, Sunanda Bhattacharyya, Mrinal Kanti Bhattacharyya

**Affiliations:** 1 Department of Biochemistry, School of Life Sciences, University of Hyderabad 28614, Hyderabad, India; 2 Department of Biotechnology and Bioinformatics, School of Life Sciences, University of Hyderabad 28614, Hyderabad, India; Virginia-Maryland College of Veterinary Medicine, Blacksburg, Virginia, USA

**Keywords:** Hsp90, co-chaperone, chaperone code, *Plasmodium*, *Trypanosoma*, *Leishmania*

## Abstract

Hsp90 is considered to be the master regulator of chaperone activity within the cellular context. In addition to aiding client maturation and maintaining protein homeostasis, Hsp90 serves various non-canonical functions in model eukaryotes: ranging from protein-trafficking into the nucleus to transcriptional regulation, from chromatin remodeling to assembly and disassembly of protein complexes during DNA repair and telomere maintenance. In performing all these trades, Hsp90 collaborates with its co-chaperones in a client-specific or function-specific manner. Hsp90 undergoes various conformational changes during its chaperone cycle, which is regulated via several post-translational modifications (PTM). Different combinations of such PTMs, known as the chaperone code, also play key regulatory roles for Hsp90 functions. Here, we examine various cellular functions of Hsp90 in protozoan parasites, particularly those that shuttle between insect host and human host, adapting to a temperature difference of at least 10°C. Our analyses reveal that most of the prominent co-chaperones are present in all these parasites, except for one that is essential in model eukaryotes. We reviewed the biochemical correlates of Hsp90 and its co-chaperone interactions and realized that the physiological significance of such interplay is largely unknown in the protozoan parasites. One striking observation is the lack of sequence conservation of the parasitic co-chaperones with their human counterparts, making them attractive drug targets. Our analyses revealed that in spite of the identification of few PTMs of parasitic Hsp90 proteins, the chaperone codes remain largely elusive.

**IMPORTANCE:**

Hsp90 is a pivotal molecular chaperone involved in maintaining proteostasis and facilitating the maturation of diverse client proteins. Beyond its canonical folding functions, Hsp90 plays non-canonical roles in nuclear trafficking, transcriptional regulation, chromatin remodeling, and DNA repair. These activities are tightly regulated through interactions with specific co-chaperones and through post-translational modifications, collectively forming the “chaperone code.” This study examines Hsp90’s role in thermal adaptation of protozoan parasites when shuttling between the insect and human hosts. Here, we summarize the canonical and diverse non-canonical functions of Hsp90 in three protozoan parasites: *Plasmodium*, *Leishmania*, and *Trypanosoma*. We highlight all the Hsp90 isoforms found in these three parasites and also illustrate all the co-chaperones and post-translational modifications of Hsp90 found to be present in these protozoan parasites. Importantly, the divergence in co-chaperone sequences from human homologs in these parasites presents a promising avenue for targeted antiparasitic drug discovery and development.

## INTRODUCTION

Protozoan parasites *Plasmodium*, *Leishmania*, and *Trypanosoma* collectively affect one-fourth of the global population despite encountering a plethora of challenges in the host environment. A major challenge for these parasites is adapting to temperature fluctuations as they transit from the insect vectors to human hosts. *Plasmodium* experiences a temperature of 25–26°C in *Anopheles* mosquitoes, *Leishmania* encounters ~22°C in sandflies, *Trypanosoma cruzi* can face temperatures up to 26°C in triatomine bugs, while *Trypanosoma brucei* can face temperatures around 24°C in tsetse flies. Upon entering the human host (37°C) and during fever episodes (~40°C), they must withstand significant thermal stress. Thus, the molecular mechanism that facilitates such thermotolerance is of paramount interest.

Heat shock proteins (HSPs) are molecular chaperones that are not only expressed constitutively during the regular cell cycle but are also induced in cells under a variety of stress conditions. Hsp90 is a highly conserved chaperone with critical roles in cell cycle regulation, cellular growth, steroid hormone signaling, and stress response pathways (1). While well-characterized in the model eukaryotes, its roles in the biology of the protozoan parasites have begun to unfold only recently. This review examines the multifaceted roles of Hsp90 and its co-chaperones in the biology of these three vector-borne protozoan parasites. This review does not include the vast literatures on the chemical inhibitors of parasitic Hsp90. *Plasmodium falciparum* is a protozoan parasite that causes the most severe and deadly form of malaria in humans. It is transmitted through the bite of infected female *Anopheles* mosquitoes, which act as the disease’s vector. *Leishmania donovani* is a protozoan parasite responsible for visceral leishmaniasis, a potentially fatal disease affecting internal organs like the liver and spleen and is transmitted to humans through the bite of infected female *Phlebotomus* sandflies. We also discuss the dynamics of Hsp90 in two different species of *Trypanosoma*, namely, *T. brucei* and *T. cruzi. T. brucei* is a protozoan parasite that causes African sleeping sickness, transmitted to humans by the bite of infected *tsetse flies*, while *T. cruzi* causes Chagas disease in the Americas and is spread through contact with the feces of infected *triatomine bugs*. Figure S1 and reference 2 illustrate the life cycle of these parasites for contextual support.

### Heat shock response in protozoan parasites

Protozoan parasites upregulate Hsp90 at both mRNA and protein levels in response to heat shock within the human host (3, 4). While this process is regulated by heat shock transcription factor 1 (HSF1) in yeast and higher eukaryotes (5), protozoan parasites lack HSF1, leaving their Hsp90 induction mechanisms unclear. Studies have identified *Plasmodium falciparum* heat shock binding protein (PfHSBP) and *P. falciparum* Apicomplexan AP2 transcription factor-heat shock (PfAP2-HS) as key regulators of Hsp90 in *P. falciparum* (4, 6). In mammals, HSBP negatively regulates HSPs’ expression, becoming inactive under stress, thereby permitting HSF activation. PfHSBP has been implicated in heat shock response (6). *Pf*HSBP is predominantly localized in the cytoplasm under normal conditions; however, upon heat stress, it gets translocated to the nucleus (5). Nuclear localization of *Pf*HSBP upon heat shock and its direct interaction with PfHsp70 is suggestive of its potential role in regulation of heat shock response (5). However, the presence of HSF1-like transcription factors in protozoan parasites should be investigated. BLAST analysis reveals an ortholog of PfHSBP in *Trypanosoma* but not in *Leishmania*. Meanwhile, PfAP2-HS is the only known transcription factor that upregulates Hsp90 in *Plasmodium*, with no equivalent factor yet identified in other protozoan parasites (4).

### Isoforms of Hsp90 in protozoan parasites

The human Hsp90 family consists of four isoforms—Hsp90α, Hsp90β, TRAP1, and Grp94—likely originating from gene duplication (7). Hsp90α is stress-inducible, whereas Hsp90β is constitutively expressed (8). Grp94, found in the ER, regulates calcium homeostasis and is retained via a KDEL sequence (9, 10). TRAP1, the mitochondrial isoform, resembles bacterial HtpG and is likely to function as a holdase under high temperatures (11). *Saccharomyces cerevisiae* has two cytosolic Hsp90 isoforms but lacks TRAP1 and Grp94 (12).

Among the protozoan parasites, *P. falciparum* harbors four Hsp90 isoforms, of which PfHsp90, PfGrp94, and PfTrap1 are inducible, and the uncharacterized isoform Hsp90_constitutive is expressed constitutively. PfHsp90 shows 77.6% sequence similarities with human Hsp90α and the structure of amino-terminal ATP binding domain (PDB: 3K60) is highly similar to human isoform (13). PfHsp90 has higher ATP-binding affinity and ATPase activity than human Hsp90, does not require Mg²^+^, and has a more hydrophobic ATP-binding pocket (14, 15). Unlike human Grp94, PfGrp94 ends with an SDEL sequence instead of KDEL (16), but its precise localization remains unclear. Recent study shows that PfGrp94 exhibits ATPase activity and can suppress aggregation of substrates. Its chaperone function was shown to be abrogated by 5′-N-ethyl-carboxamide-adenosine, and the inhibitor displays moderate anti-plasmodial activity against NF54, K1, and Dd2 strains (17). Crystal structure of PfTRAP1 reveals that dynamic conformational changes in domain I are essential for ligand binding, gliding motility, and sporozoite transmission from mosquito to mammals (18).

In *Leishmania major*, Hsp83 exists in multiple tandem copies (~17 per genome), contributing to high expression (19). LdHsp83 shares 62.57% sequence identity with human Hsp90α, while putative isoforms LmTrap and LmGrp remain uncharacterized. In *T. brucei*, Hsp83 occurs in 10 tandem copies (20, 21), shares 61.45% identity with human Hsp90α, and has a higher ATP affinity than its human counterpart (22). TbHsp83 is essential for cytokinesis, while TbTRAP1 (TbHsp84) plays a role in kinetoplast DNA (kDNA) replication (23), though the function of TbGrp94 remains unknown. *T. cruzi* also encodes three different isoforms of Hsp90: TcHsp83, TcGrp94, and TcTrap1. Here, we list all the known, putative, and experimentally or biochemically characterized isoforms of Hsp90 in these protozoan parasites (Table S1). A schematic comparison of Hsp90α orthologs across protozoan parasites, human, and yeast is illustrated in Fig. S2.

## CANONICAL AND NON-CANONICAL FUNCTIONS OF Hsp90

**Fig 1:**
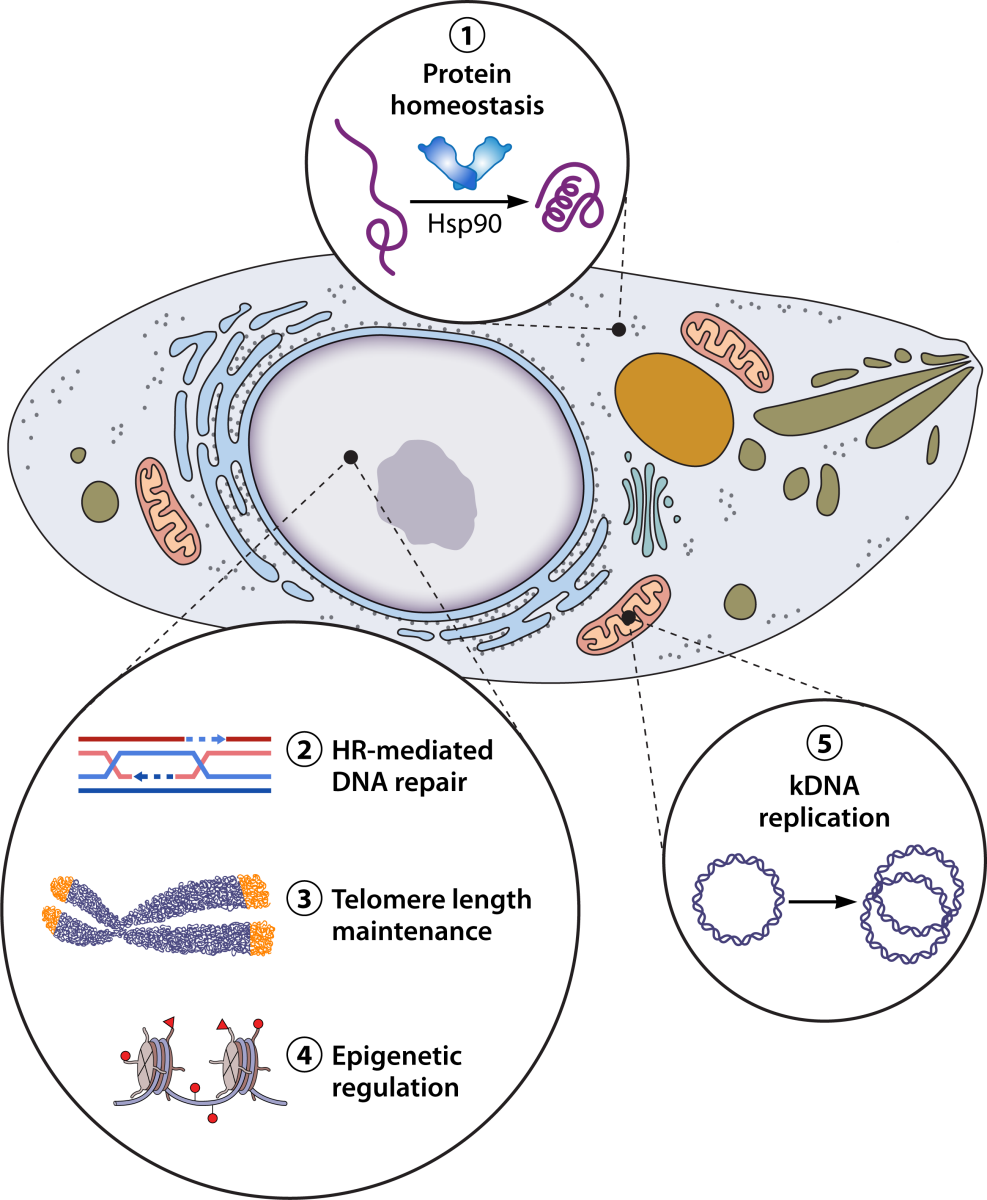
Schematic depicting the canonical and non-canonical functions performed by Hsp90 in a parasitic protozoan. Apart from its canonical function of protein homeostasis, Hsp90 is seen to be important for DNA repair, telomere length maintenance, epigenetic regulation, and mitochondrial DNA (kDNA) synthesis in vector-borne protozoan parasites.

In addition to the primary or the canonical function Hsp90, over the past decade non-canonical moonlighting functions of Hsp90 have emerged. Here, we summarize the current knowledge on the functions of Hsp90 of *Plasmodium*, *Trypanosoma*, and *Leishmania* parasites. Figure 1 highlights the different pathways that require the Hsp90 chaperone including protein homeostasis, HR-mediated DNA repair, telomere length maintenance, epigenetic regulation, and mitochondrial DNA (kDNA) replication. We shall discuss below the involvement of Hsp90 in these pathways.

### Canonical function of Hsp90: maintenance of protein homeostasis

Structural variations among the Hsp90 isoforms contribute to functional differences among its paralogs and orthologs. All Hsp90 homologs contain three primary structural domains: the N-terminal nucleotide-binding domain (NTD), the middle domain (MD), and the C-terminal domain (CTD) (24). Hsp90 facilitates protein folding through ATP-dependent conformational changes, where ATP binding and hydrolysis occur via interactions between the NTD and MD (24, 25). A charged linker region connects the NTD and MD, playing a key role in inter-domain communication and overall chaperone function (25).

In *P. falciparum*, PfHsp90 maintains the parasite’s 26S proteasome complex by directly promoting proteasome hydrolysis and chaperoning the active 26S complex (26). Additionally, PfHsp90 interacts with PfRad51, a crucial homologous recombination (HR) protein involved in DNA repair. Inhibition of PfHsp90 results in the degradation of PfRad51 via the proteasomal pathway, leading to impaired DNA repair and increased sensitivity to DNA-damaging agents (27). The inhibition of PfHsp90 by 17-AAG in combination with PfRad51 inhibitor B02 has been shown to have a synergistic effect, suggesting PfHsp90 plays a critical role in the parasite’s DNA double-strand break repair mechanisms (27). Another key client of PfHsp90 is the histone deacetylase PfSir2A, which is involved in epigenetic gene regulation. Disruption of PfHsp90 activity impairs PfSir2A function, leading to the de-repression of subtelomeric *var* genes (28).

While significant progress has been made in understanding PfHsp90’s client interactions, no direct Hsp90 client proteins have been identified in *Trypanosoma* or *Leishmania* to date. Further research is required to determine the functional roles and client interactions of Hsp90 in these protozoan parasites.

### Non-canonical function of Hsp90: maintenance of genome stability

Hsp90 is known to contribute to genome stability in protozoan parasites by facilitating DNA repair in malaria parasites, maintaining telomere length in *Leishmania*, and supporting mitochondrial DNA replication in *Trypanosoma*.

#### DNA repair

It has recently been reported that nuclear translocation of yHSP90α and its recruitment to DNA double-strand breaks is important for HR-mediated DNA repair in yeast (29). PfHsp90 is known to physically interact with PfRad51 and simultaneously promotes the UV irradiation-induced DNA repair activity of PfRad51, the key DNA recombinase (27). However, in addition to providing stability to PfRad51, whether Hsp90 has any non-canonical role during the assembly of the repair proteins on the damaged chromosomes remains elusive. In higher eukaryotes, another nuclear function of Hsp90 includes the formation of γH2Ax foci at the damaged chromosomes (30). These reports intrigue us to wonder whether the DNA damage response is regulated by this master chaperone in *Plasmodium*, *Leishmania*, and *Trypanosoma*.

#### Telomere length maintenance

Inhibition of LaHsp90 in *Leishmania amazonensis* by 17AAG resulted in telomere shortening and inhibition of telomerase activity (31). Hsp90 is known to perform a similar function in yeast and mammals where it promotes the telomerase-DNA association by assembling active telomerase (32). The interaction between Hsp90 and telomerase in *Plasmodium* and *Trypanosoma*, if any, is yet to be established.

#### Mitochondrial DNA replication

Knockdown of mitochondrial Hsp90 (Hsp84) in *T. brucei* is seen to halt the synthesis of new kDNA generating cells that are devoid of kDNA (23). However, the precise function of Hsp90 in kDNA synthesis still remains elusive and needs to be studied further in other kinetoplast parasites.

### Non-canonical function of Hsp90: epigenetic regulation of gene expression

PfHsp90 has also been seen to act as a transcriptional down regulator of two histone deacetylases, *PfSIR2A* and *PfSIR2B*, as a response to febrile temperatures (33). This results in a reduced occupancy of PfSir2 at the subtelomeric *var* promoters that leads to de-repression of *var* genes and allows the parasite to manipulate the expression of its epigenetic modifiers. Such regulation is yet to be explored in *Leishmania* and *Trypanosoma*. Such Hsp90-mediated regulation of Sir2 proteins in other protozoan parasites has not been studied so far.

## MODULATION OF Hsp90 FUNCTION BY ITS COCHAPERONE PLASTICITY

Hsp90 is known to be regulated by a cohort of co-chaperones (34). Co-chaperones mediate the interaction between Hsp90 and other chaperones in order to promote client-protein transfer. They also control conformational changes of Hsp90 by regulating its ATPase activity and mediate its interaction with client proteins. Hsp90, however, can’t act on the substrate proteins that are completely unfolded; instead, it works on the partially folded substrates in the later stage of protein folding. Hsp70 collaborates with its cochaperones, namely, J domain protein (DnaJ/Hsp40) and nucleotide exchange factor (NEF) to convert nascent polypeptide chains into the partially folded state (Fig. S3). In this process, the Hsp70 interacting protein (HIP) decelerates ADP dissociation from Hsp70 and, thereby, stabilizes Hsp70-substrate complex. Thus, NEF and HIP exert opposing effects on Hsp70 and are mutually exclusive ( 35). Hsp70-Hsp90 organizing protein (HOP) facilitates the transfer of clients to the Hsp90 chaperon system. Both Hsp70 cycle and Hsp90 cycle are assisted by several cochaperones. In this article, we focus only on the cochaperones of Hsp90.

It has been experimentally validated that the requirement of a specific cochaperone is dictated by its respective client protein (34). While Hsp90 in protozoan parasites is a potential drug target, its high sequence homology with human Hsp90 limits selectivity. However, parasite co-chaperones, which show low sequence similarity to human homologs, offer promising drug targets.

Co-purification of Hsp90 complexes obtained from yeast or mammalian cell extracts allowed for the initial identification of the majority of Hsp90 co-chaperones (particularly Hsp70-Hsp90 organizing protein [Hop], protein phosphatase 5 [PP5], p23, Sgt1, FKBP51/52, cyclophilin 40 [Cyp40], and cell division cycle 37 [Cdc37]) (36). Additional co-chaperones, TPR-containing protein associated with Hsp90 (Tah1), activator of Hsp90 ATPase (Aha1), and Cns1, were discovered as a result of their genetic or physical interactions with *S. cerevisiae’s* Hsp90 (36). The largest family of Hsp90 co-chaperones is the tetratricopeptide repeat (TPR)-containing proteins, although not all TPR-containing proteins have been analyzed for co-chaperone activity. TPR co-chaperones do not directly bind to the non-native proteins but have an integral role in directing the activity of Hsp90. Transfer of a client protein from Hsp70 to Hsp90 is facilitated by the TPR containing protein Hop (Yeast homolog Sti1), which is able to simultaneously bind with Hsp70 and Hsp90 through separate TPR domains (37). Displacement of Hop by other TPR co-chaperones promotes ATP binding and conformational changes. P23 stabilizes the closed Hsp90 conformation, while Cdc37 specializes in kinase folding (38). The low intrinsic ATPase activity of Hsp90 is accelerated by the Aha1, which regulates client association (39, 40). Amino-terminal domain of Aha1 binds to the MD of one Hsp90 protomer to modulate the ATP hydrolysis cycle, so that the clients are released in a timely manner. The number of identified human Hsp90 co-chaperones has grown from 6 to 50 (41), reflecting its diverse roles. In contrast, parasite Hsp90 co-chaperones remain less studied. *P. falciparum* has over 10 predicted co-chaperones, but only five (Pfp23, PfAha1, PfPP5, PfHop, and Pf FK506-binding protein 35 [FKBP35]) have been characterized (42–49). It was observed that PfHop, Pfp23, and PfAha1 are also found in *Leishmania* and *Trypanosoma* (Table S2).

### Hsp70-Hsp90 organizing protein

Hop facilitates client protein transfer between Hsp70 and Hsp90 (50). In *P. falciparum*, a single-copy co-chaperone, PfHop (PF14_0324), shares 37.68% and 34.23% sequence identity with human Hop and yeast Sti1, respectively. Its association with PfHsp70 and PfHsp90 was suggested through co-immunoprecipitation (45). PfHop co-localizes with PfHsp70 and PfHsp90 in *P. falciparum* trophozoites, indicating that the protein may mediate functional interaction between the two cochaperones as seen in other organisms (45). Structural analysis reveals PfHop contains three TPR domains, with conserved residues in TPR1 (K11 and N15) and TPR2A (K247, N251, and K319) important for Hsp70 and Hsp90 binding, respectively (51). A yeast two-hybrid study identified PfHop’s interaction with Falcipain 2B, a cysteine protease involved in hemoglobin degradation, suggesting PfHsp90-PfHop1’s role in hemoglobin metabolism in malaria parasite (52).

In *Leishmania*, Hop proteins (LbrM.33.0350 and LmjF08.110) show 35-39% identity with Sti1 and HsHop, respectively (53). Hop/Sti1 interaction with Hsp83 is essential for *Leishmania* viability (54, 55). LbHop interacts with LbHsp90 via its TPR2A-TPR2B domains (53). In *L. donovani*, LdHsp90 co-localizes with LdSti1, and disruption of their interaction reduces parasite load by 30%, likely due to impaired amastigote proliferation (56).

Using BLAST-P, we determined that *T. cruzi* STI-1 ortholog (AF107772.1) shows 39.67% identity with its human counterpart and it has been shown to interact with Hsp83 and Hsp70 (57). TcSti1 is upregulated during nutritional stress and is critical for parasite differentiation (57).

### Activator of Hsp90 ATPase

Genomic analysis identifies two *P. falciparum* Aha1 isoforms PF3D7_0306200 and PF3D7_0308500, and they show 28.95% and 30.30% sequence identity with the human counterpart, respectively. PF3D7_0306200 isoform has been characterized, and it is known to stimulate PfHsp90’s ATPase activity threefold (43). A glutathione S-transferase (GST) pull-down assay demonstrated that PfAha1 interacts with PfHsp90 in an MgCl₂ and ATP-dependent manner, competing with Pfp23 for binding (43). Bio-computational modeling and mutagenesis identified residue N108 crucial for PfHsp90 interaction (43). The presence of PfAha1 suggests that despite the higher basal ATPase activity of PfHsp90 compared to hHsp90, client release from chaperone complexes at the later stage of folding might be regulated by Aha1-mediated ATPase stimulation. There is no report about the putative PfAha1, PF3D7_0308500, so far.

In *Leishmania braziliensis*, Aha1 interacts with Hsp90, increasing ATPase activity ~10-fold (58), while *L. donovani* Aha1 is constitutively expressed, interacting with Hsp90 and being involved in ethanol tolerance and infectivity (59).

Proteomic studies in *T. brucei* have shown that TbbAha1 is upregulated during the bloodstream form (BSF) (60–62) stage and is crucial for the parasite’s survival at this stage (63).

### p23

The *P. falciparum* p23 co-chaperone (Pfp23; PF3D7_1453700) was first identified as a phosphoprotein highly expressed in the trophozoite stage (64). This isoform, renamed as p23B, interacts with PfHsp90 in an ATP-dependent manner (42), with conserved residues K91, H93, W94, and K96 being crucial for this interaction (42). A second acidic isoform, Pfp23A (PF3D7_0927000), has also been identified (48). Our BLAST-P analysis shows that Pfp23A and Pfp23B share 24% and 29.17% identity with human p23, respectively. The presence of two different Pfp23 isoforms in *P. falciparum* with putative functional differences is interesting and suggests that the mechanism of stabilization of PfHsp90 late-stage complexes might differ from that of the human Hsp90 complex. Both isoforms inhibit PfHsp90’s ATPase activity, with Pfp23A being more effective, exhibiting higher thermal stability and intrinsic chaperone activity independent of PfHsp90 (42, 48). By slowing ATPase activity, p23 retains Hsp90 in an ATP-bound state, prolonging client protein interaction for activation (65).

Similarly, *L. braziliensis* has two p23 isoforms (Lbp23A; XP_001568545.1 and Lbp23B; XP_001564309.1), sharing 30% identity with each other and with human p23 (66). Both interact with LbHsp90 and inhibit its ATPase activity, though with varying efficiencies. Structural analyses show both exist as elongated monomers, but Lbp23A has greater stability than Lbp23B, suggesting functional differences (66).

In *T. brucei*, Tbp23a (Tb927.9.10230) and Tbp23b (Tb927.10.2620) share 28% identity and exhibit 33% and 26% identity with human p23, respectively (20). RNAi-mediated knockdown experiments reveal that each p23 protein is critical for parasite survival during distinct stages of its life cycle (63). Further studies are needed to fully characterize their distinct roles.

### CyPs: Cyp40 and FKBP35

Immunophilins, including cyclophilins (CyPs) and FK506-binding proteins (FKBPs), function as peptidyl-prolyl *cis-trans* isomerases (PPIases) crucial for protein folding, similar to HSPs. Some of the CyPs (human Cyp40 and yeast Cpr6/Cpr7) and FKBPs (human FKBP36, FKBP37, FKBP38, FKBP51, and FKBP52) contain additional TPR domains at their CTD, with which they interact with Hsp90 and regulate its client folding (67, 68). These domains interact with Hsp90’s EEVD motif via dicarboxylate clamp interactions (69).

In *P. falciparum*, eight CyPs exhibit chaperone-like activity, with PfCyp19A and PfCyp19B showing PPIase activity as well and found to be inhibited by cyclosporin A with IC_50_: 10 and 15 nM, respectively (47). Though lacking the *P**lasmodium* export element motif, PfCyp19B localizes to infected erythrocyte surface, potentially aiding virulence (70). However, none of the eight CyPs possesses the signature TPR domain, which is required for Hsp90 binding, and PfFKBP35 (*PF3D7_1247400*) is the only *P. falciparum* immunophilin with a TPR domain, which binds to PfHsp90 and exhibits strong PPIase activity and is sensitive to inhibition by ascomycin and rapamycin (71, 72). PfFKBP35, being the only cochaperone harboring TPR domain, qualifies as an important antimalaria drug target. Its knockdown impairs ribosomal protein levels and protein synthesis, causing a delayed-death phenotype (73). Interestingly, it was observed that FK506 displayed parasiticidal activity in a PfFKBP35-independent manner, indicating putative FK506 targets beyond PfFKBP35. Studies have been done to synthesize potent inhibitors of PfFKBP35-PPIase domain, which shows better selectivity compared to human PPIase. According to X-ray and nuclear magnetic resonance (NMR) crystallographic investigations, the FK506 binding pocket of PfFKBP35 is structurally very similar to that of human FKBP12, except for few unique amino acids that are specific in *Plasmodium* (72). These differences were employed in the development of inhibitors that can specifically inhibit PfFKBP35. The structural variations were found in the β5–β6 segments of the PPIase domain, in which PfFKBP35 has a conserved cysteine and serine residue at amino acid positions 106 and 109, respectively, whereas human FKBP12 has a histidine (H87) and an isoleucine (I90) residue at the same position. In order to achieve selectivity toward PfPFBP35, small molecules were designed targeting the conserved C106/C105 and S109/S108 residues in PfFKBP35/PvFKBP35 (*Plasmodium vivax* FKBP35) (74). A novel inhibitor, D44, was shown to target PfFKBP35 with >100-fold selectivity over human homologs (75, 76).

The first PPIases identified in *L. major* and *L. donovani* were CyPs, discovered during research using cyclosporin A (77, 78). These CyPs often include extra N- or C-terminal extensions in addition to the core CyP-like domain (CLD) (79). Notable examples include LmCyP24.6 (LmaCyP5), which has a prokaryotic lipid attachment domain (PLD); LmCyP38 (LmaCyP40), which contains two TPR domains; and LdCyP38.4 (LdCyP40), featuring a single TPR domain at the C-terminus. Similarly, FKBPs found in both species contain the conserved FKBP PPIase domain. In *L. donovani*, LdCyP (AAD46565.1) shares biochemical properties with human CyPs, aiding protein refolding (80). LdCyp40 expression peaks in amastigotes, and knockout mutants display cytoskeletal defects and fail to establish infections (81). *Leishmania* encodes 17 CyP-like and five FKBP-like proteins, with LmCyp40 binding cyclosporin A, which disrupts proliferation without direct cytotoxicity (82).

In *T. brucei*, genome analysis identified Cyp40 (XP_827280.1) and a putative FKB-506 binding-like protein (FKBPL/XP_828079.1) (20). Among the *T. cruzi* CyPs, TcCyP21 is the only one with a resolved crystal structure (PDB: 1XO7) (83). Several CyPs, including TcCyP22, TcCyP26, TcCyP30, and TcCyP42, have extended N-terminal regions, while TcCyP20 features a small extension at the C-terminus (84). CyPs such as TcCyP25, TcCyP28, TcCyP29, TcCyP35.3, and TcCyP35 (formerly TcCyP34) exhibit elongated segments on both termini (85). Additionally, TcCyP38 (formerly TcCyP40) includes a TPR domain, and the largest CyP, TcCyP103 (formerly TcCyP110), possesses disordered structural regions rich in both basic and acidic residues flanking its CLD domain (85). The significance of such disordered regions or the N- or C-terminal extensions is currently unknown.

### Pih1 and Tah1

The maturation of small nucleolar ribonucleoprotein particles (snoRNPs) involves two specialized Hsp90 cochaperones: protein interacting with Hsp90 (Pih1) and Tah1 (TPR-containing protein associated with Hsp90) (86, 87). These proteins form a heterodimeric complex that acts as an adapter to recruit client proteins, bridging them with Hsp90 (88). In humans, Tah1, known as RNA polymerase II associated protein 3 (RPAP3 ), has two TPR domains that interact with the EEVD motifs of both Hsp90 and Hsp70 and also stabilizes Pih1. Pih1 binds phosphorylated clients via its amino-terminal domain and interacts with Tah1 through its CS domain (CHORD domain containing protein and Sgt1 domain). Genome-wide yeast studies identified the AAA(+)-type DNA helicases Rvb-1 and Rvb-2 as potential interaction partners of Tah1/Pih1, forming the R2TP complex (86, 88). The R2TP-Hsp90 complex plays a role in snoRNP biogenesis, RNA polymerase II assembly, and epigenetic gene regulation.

To identify a Pih1 homolog in *P. falciparum*, a BLAST-P analysis revealed a high-scoring protein (Pf3D7_1235000) with 27% sequence identity to human Pih1 but with a longer amino acid sequence (89). Sequence analysis showed that PfPIH1D1 contains a CS domain and an additional C-terminal tail, though no experimental studies have been conducted. Similarly, a search for Tah1 orthologs *in P. falciparum* identified two proteins (Pf3D7_1434300 and Pf3D7_0213500) containing TPR repeats, with Pf3D7_1434300 showing greater similarity to human RPAP3 (89). However, no experimentally verified RPAP3 protein has been reported in *Plasmodium*, and the interaction between PfPIH1D1 and PfRVBL proteins remains unknown.

In *L. donovani*, Pih1 homolog (LdPih1, Ldbpk_354400.1) was identified sharing 31% identity with the human protein (89). Additionally, a Tah1-like protein (Ldbpk_081020.1) with characteristic TPR motifs was identified, showing 28% sequence identity with human RPAP3 (89). Phylogenetic analysis indicates that *L. donovani* Tah1 is more closely related to human RPAP3 than to yeast Tah1 (89). Moreover, a novel TPR-domain-containing gene, LmTPR, conserved in *Leishmania*, has been recently characterized (90). LmTPR is expressed in all developmental stages, and binding assays confirmed its interactions with both Hsp90 and Hsp70 (90). Sequence predictions suggest that LmTPR contains a C-terminal RPAP3 domain, but further studies are needed to determine whether LmTPR-Hsp90 interacts with LmPih1.

In *T. brucei*, the Pih1 ortholog is TbPih1, Tb927.9.10490. The presence of a Tah1-like protein in *Trypanosoma* remains unclear. Data mining identified the Pih1 ortholog in *T. cruzi* and *L. major* as a putative pre-rRNA processing protein, also known as Nop17, while in *T. brucei*, the ortholog is annotated as CMF56**,** a component of motile flagella (20). Proteomic data confirmed the expression of PIH1D3 in both the bloodstream and procyclic stages of the parasite (20).

### Protein phosphatase 5

PP5, a serine/threonine phosphatase, regulates Hsp90 activity by binding to its EEVD motif via its TPR domain, relieving autoinhibition and enhancing phosphatase function (91–93). In yeast, PPT1 deletion results in Hsp90 hyperphosphorylation and reduced chaperone efficiency (94). PP5 also dephosphorylates Cdc37 at S13, affecting kinase activation in Hsp90 complexes(95).

Our BLASTp analysis revealed PfPP5 (PF3D7_1355500) shares 43.64% sequence identity with human PP5 but has four TPR domains instead of three (44). It is expressed across all intraerythrocytic stages and interacts with PfHsp90, but its role in PfHsp90 dephosphorylation remains unclear (44).

In *Leishmania*, LmPP5 (XP_001682421) shares 46% identity with human PP5 and contains three TPR motifs and was shown to have phosphatase activity (96). The gain-of-function mutation E51A on the TPR domain of LmPP5 increased LmPP5 activity, suggesting a potential inhibitory role of this residue. LmPP5 expression peaks in metacyclic promastigotes, and PP5 knockout mutants show Hsp83 hyperphosphorylation and impaired stress adaptation, though infectivity remains unaffected (96).

In *T. brucei*, TbPP5 (Tb927.10.13670) has three TPR repeats and 49.15% sequence identity with human PP5 (97). Recombinant TbPP5 exhibits phosphatase activity, stimulated 2.6-fold by arachidonic acid (97). It is more expressed in procyclic than BSFs and peaks in G1 phase (98). TbPP5 knockdown impairs bloodstream-form growth, while overexpression supports growth in serum-deprived conditions, suggesting a role in serum factor-mediated signaling (98). TbPP5 and Hsp90 translocate to the nucleus under heat stress or geldanamycin (GA) treatment, and TbPP5-deficient cells are more sensitive to GA (99).

### SGT1/calcyclin-binding protein (CBP)

Sgt1 functions as a client protein adapter, recruiting diverse clients to the Hsp90 chaperone system for maturation. It consists of three domains: a TPR domain at the N-terminal, a CS domain in the middle, and an SGS domain at the C-terminal. It has been established that Sgt1 binds to Hsp90 through its CS domain instead of the TPR domain (100).

A BLAST-P analysis identified a putative Sgt1/CBP co-chaperone, *PfCBP* (PF3D7_0933200), in *P. falciparum*. It was found that *PfCBP* shares 44.83% sequence identity with its human counterpart, but further studies are needed to confirm its interaction with *PfHsp90* and its role in parasite physiology. An atypical SGT-like protein has been identified in the *L. donovani* genome that lacks the characteristic glutamine-rich region at its C-terminus (101). The gene is expressed constitutively and is essential for survival or growth (101). It co-localizes with HIP in the cytoplasm and partially overlaps with Hsp70 and Hsp90, indicating possible interactions with both chaperones (101). In *L. donovani*, the Sgt ortholog is essential for promastigote growth and survival. LdSgt was shown to assemble into large, stable complexes with molecular chaperones such as Hsp83, Hsp70, HIP, HOP, J-proteins, and Hsp100 (101), while the recombinant Sgt from *L. braziliensis* was found to interact with both LbHsp90 and human Hsp70-1A (102). Proteomic studies revealed that TbbSGT is upregulated in the procyclic stage of *T. brucei* and is present in both the flagellar and cell surface proteomes (60–62, 103, 104). These findings suggest that the Sgt ortholog in *T. brucei* may serve similar functions, potentially contributing to the assembly of the Hsp83 chaperone machinery in *T. brucei*. However, further research is needed to clarify the interactions between Sgt and Hsp70/Hsp83 in *T. brucei*.

### CHIP

Co-chaperones regulate protein homeostasis by facilitating both protein folding and degradation. Carboxy-terminus of Hsp70-interacting protein (CHIP) possesses a unique structure with both a U-box RING finger domain and a TPR domain. The U-box enables CHIP to function as an E3 ubiquitin ligase, directing proteins for proteasomal degradation while still bound to Hsp90 and Hsp70 via its TPR domain. CHIP engagement leads to client protein ubiquitination and subsequent degradation (105, 106).

The competition between CHIP and Hop for Hsp90 binding determines a protein’s fate: CHIP binding promotes degradation, particularly when Hsp90 is unphosphorylated, whereas Hop favors folding when bound to phosphorylated Hsp90α (106). Thus, C-terminal phosphorylation of Hsp90α acts as a switch for cochaperone binding. Although CHIP is a critical Hsp90 co-chaperone, no homolog has been found in yeast. Bioinformatics analysis predicts the presence of *PfCHIP* (PF3D7_0527500) (107) in the *Plasmodium* genome, with BLAST-P analysis revealing 24% identity to its human counterpart. However, CHIP remains unidentified in *Leishmania* and *Trypanosoma*.

### Cdc37 homolog potentially missing in protozoan parasites

Cdc37 is essential for activating several protein kinases, including Cdk1, Cdk4, Akt, v-Src, Raf, and CK2 (108, 109). It facilitates the recruitment of nascent or unstable kinases to Hsp90 for proper folding and activation (110, 111). Notably, Cdc37 stabilizes up to 65% of yeast kinases (112) and mediates the interaction of 60% of human kinases with Hsp90 (113).

Interestingly, no homolog of Cdc37 has been identified in *Plasmodium*, *Leishmania*, or *Trypanosoma*. It is hypothesized that other *P. falciparum* co-chaperones may compensate for its absence, as essential kinases such as Cdk1 (*PfPK5*), Akt (*PfPKB*), and CK2 (*PfCK2*) are present in *Plasmodium* genome. The failure to identify a Cdc37 ortholog could either be due to significant sequence divergence or alternative chaperone mechanisms in these parasites.

### Fine-tuning of Hsp90 function through PTMs

Hsp90’s function is modulated by ATP binding, hydrolysis, co-chaperone interactions, and post-translational modifications (PTMs) such as phosphorylation, acetylation, SUMOylation, methylation, ubiquitination, and O-GlcNAcylation (114). These PTMs regulate Hsp90’s interactions with co-chaperones and client proteins.

In *P. falciparum*, acetylation of PfHsp90 at Lys-381 and Lys-426 within the p23 and Aha1 binding domains suggests a role in multi-chaperone complex assembly (115). Inhibition of histone deacetylases partially disrupted PfHsp90 complexes, highlighting acetylation’s functional significance (115).

Phosphorylation is a key PTM in *L. donovani* Hsp90, with Thr223 and Ser526 identified as modification sites (55). Thr223 is analogous to a serine in human Hsp90, whereas Ser526 is parasite-specific, indicating a potential regulatory function in *Leishmania*, *Plasmodium*, and *Trypanosoma*.

In *T. brucei*, phosphorylation sites T211, T216, S597, and S694 are conserved across kinetoplastids, while S374 is shared by *Plasmodium*, *Leishmania*, and humans. Acetylation sites (K44, K227, K289, K394, K421, and K515), N-glycosylation sites (N90, N372, N612), and ubiquitination sites (K394, K560) have also been reported (20).

In humans, PTMs regulate Hsp90 in various cellular processes. Acetylation at K69, K100, and K558 promotes extracellular export, while phosphorylation at T90 reduces ATP binding and Y197 disrupts Hsp90:Cdc37 interactions (114).

**Fig 2:**
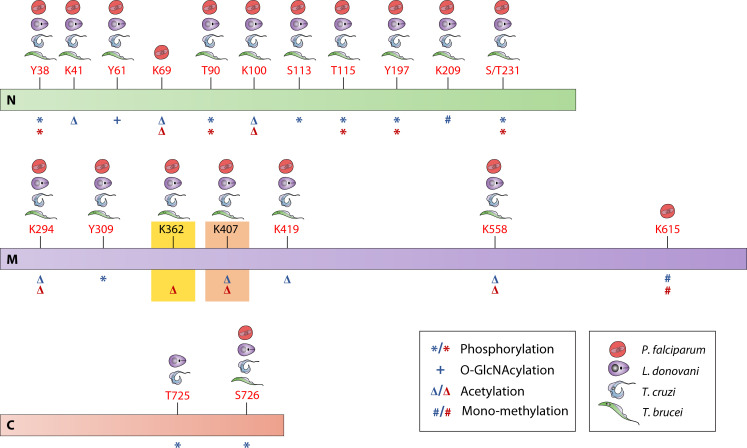
Schematic depicting the PTMs of Hsp90 in vector-borne protozoan parasites. The top bar (green) represents the N-terminal domain, the middle bar (purple) represents the MD, and the bottom bar (orange) represents the CTD of Hsp90. Human Hsp90 has been taken as the reference scale here. Conserved amino acid residues that are present and predicted to be modified in any of the given three parasites have been represented in red, and those which are experimentally validated are shown in black. The type of modification is mentioned below each residue using special characters where＊represents phosphorylation, + represents O-GlcNAcylation, Δ represents acetylation, and # represents mono-methylation. Characters in blue represent the role of that modification in cellular function(s), while orange-colored characters represent a role in chaperone binding. The conservation of each stated residue in any of the three parasites has been represented by respective parasite symbols (as mentioned in the key). The modification highlighted in yellow is experimentally validated in *Plasmodium*, while the one highlighted in orange is experimentally validated in *Leishmania*.

We performed a multiple sequence alignment to identify potential residues that are conserved in *P. falciparum*, *L. donovani*, *T. cruzi*, and *T. brucei* and that show an overlap with the post-translationally modified residues that have been functionally studied in hHsp90 (Fig. 2). Conserved PTM sites across protozoan parasites suggest shared regulatory mechanisms. On the other hand, PTMs that are conserved only among parasites, such as K362, which has been experimentally validated in *Plasmodium*, could offer potential drug targets as this PTM is absent in hHsp90. However, further research is needed to elucidate their roles.

## ROLES OF Hsp90 CHAPERONE MACHINERY IN BIOLOGY OF PARASITISM AND DRUG RESISTANCE

### Cellular growth and differentiation

Hsp90 regulates developmental processes by modulating protein kinases and transcription factors critical for growth and differentiation (5). Protozoan parasites depend on Hsp90 for various life cycle transitions. In *P. falciparum*, GA-mediated inhibition of PfHsp90 disrupts multiple stages, including ring-to-trophozoite transition, schizont development, merozoite release, and reinvasion, corresponding with peak Hsp90 synthesis during ring-to-trophozoite transition (116). In *L. donovani*, LdHsp90 is essential for promastigote-to-amastigote differentiation, with GA treatment inducing G2-phase arrest (117). Similarly, in *T. cruzi*, GA treatment results in growth arrest and blocks trypomastigote-to-epimastigote differentiation, underscoring TcHsp83’s role in protein maturation required for epimastigote differentiation (118).

### Fertilization

Hsp90 plays a crucial role in *Plasmodium berghei* fertilization by acting as a ligand on the surface of female gametes, facilitating male-female gamete interaction and oocyst formation during the parasite’s sexual life cycle (119). Anti-Hsp90 antibodies strongly inhibit *P. berghei* oocyst development in mosquitoes, suggesting its potential as a transmission-blocking target (119). Insertional inactivation of TRAP1 in *P. berghei* demonstrated the critical role of PbTRAP1 in sporozoite infection of the mosquito salivary glands and essential for sporozoite gliding motility (120). The role of Hsp90 in sexual stages of development among other protozoan parasites remains unknown.

### Virulence/pathogenesis

Hsp90 plays a crucial role in the pathogenesis of protozoan infections. In *Leishmania*, the endoplasmic reticulum variant of Hsp90, glucose-regulated protein 94 (GRP94), regulates virulence by influencing the synthesis of lipophosphoglycan (LPG), a known virulence factor (121, 122). The inactivation of GRP94 leads to reduced LPG synthesis, ultimately decreasing parasite virulence (121). Additionally, malaria parasites exhibit compensatory overexpression of Hsp90 and its co-chaperones (Hsp70, Hsp27, gp96, etc.) in response to pharmacological inhibition, which enhances parasite survival under adverse conditions (123).

## FUTURE PERSPECTIVES

The Hsp90 chaperone system in protozoan parasites is a promising drug target. However, most studies rely on repurposed drugs originally designed for human Hsp90. Despite some inhibitors showing selectivity for the parasitic protein, drug toxicity remains a significant concern. At this end, the parasitic co-chaperones could offer the much-needed breakthrough, primarily because of the less sequence conservation with their human counterparts. A second feature of the parasitic co-chaperones qualifies them as better candidates as drug targets. It appeared that all the parasites contain a reduced number of co-chaperone proteome compared to the yeast or human, emphasizing a much-reduced functional redundancy. However, the co-chaperone requirement for each biological function of parasitic Hsp90 is largely unknown. The advancement of CRISPR-mediated knockout or other knockdown strategies in these parasites will now enable the study of the functional roles of the co-chaperones in both the canonical and non-canonical functions of Hsp90. The absence of CDC37 in all the parasites is highly interesting, given that most kinase clients of Hsp90 require CDC37 as a co-chaperone, and as a result, *cdc37* null yeast strains are inviable. As all the parasites possess a large number of kinases, it will be important to identify the co-chaperones that perform a CDC37-like job in these parasites. Currently, research activities aiming identification of the Hsp90 chaperone code for each biological processes have taken center stage. Such work in protozoan parasites is in its infancy. Extensive research on the chaperone code in these protozoan parasites will not only advance our fundamental understanding of Hsp90 chaperone machine but also will open up innovative ways to target parasitic Hsp90 functions.

## SUPPLEMENTAL MATERIAL

Figure S1 msphere.00329-25-s0001.tif Life cycles of *P. falciparum*, *L. donovani*, and *T. brucei*.

Figure S2 msphere.00329-25-s0002.tif Domain architecture for different Hsp90⍺ orthologs.

Figure S3 msphere.00329-25-s0003.tif Schematic diagram depicting client maturation through Hsp90 chaperone cycle.

Legends msphere.00329-25-s0004.docx Legends for Fig. S1 to S3.

Supplemental tables msphere.00329-25-s0005.docx Tables S1 and S2.

ASM does not own the copyrights to Supplemental Material that may be linked to, or accessed through, an article. The authors have granted ASM a non-exclusive, world-wide license to publish the Supplemental Material files. Please contact the corresponding author directly for reuse.

## References

[1] Jackson SE. 2012. Hsp90: structure and function, p 155–240. In Jackson S (ed), Molecular chaperones. Springer Berlin, Heidelberg, Heidelberg.

[2] CDC - DPDx - American trypanosomiasis. 2025. Available from: https://www.cdc.gov/dpdx/trypanosomiasisamerican/index.html. Retrieved 13 Jul 2025.

[3] Prasanna P, Upadhyay A. 2021. Heat shock proteins as the druggable targets in leishmaniasis: promises and perils. Infect Immun 89:e00559-20. doi:10.1128/IAI.00559-20 33139381 PMC7822131

[4] Tintó-Font E, Michel-Todó L, Russell TJ, Casas-Vila N, Conway DJ, Bozdech Z, Llinás M, Cortés A. 2021. A heat-shock response regulated by the PfAP2-HS transcription factor protects human malaria parasites from febrile temperatures. Nat Microbiol 6:1163–1174. doi:10.1038/s41564-021-00940-w 34400833 PMC8390444

[5] Prodromou C. 2016. Mechanisms of Hsp90 regulation. Biochem J 473:2439–2452. doi:10.1042/BCJ20160005 27515256 PMC4980810

[6] Sayeed SK, Shah V, Chaubey S, Singh M, Alampalli SV, Tatu US. 2014. Identification of heat shock factor binding protein in Plasmodium falciparum. Malar J 13:118. doi:10.1186/1475-2875-13-118 24674379 PMC3994269

[7] Gupta RS. 1995. Phylogenetic analysis of the 90 kD heat shock family of protein sequences and an examination of the relationship among animals, plants, and fungi species. Mol Biol Evol 12:1063–1073. doi:10.1093/oxfordjournals.molbev.a040281 8524040

[8] Millson SH, Truman AW, Rácz A, Hu B, Panaretou B, Nuttall J, Mollapour M, Söti C, Piper PW. 2007. Expressed as the sole Hsp90 of yeast, the alpha and beta isoforms of human Hsp90 differ with regard to their capacities for activation of certain client proteins, whereas only Hsp90beta generates sensitivity to the Hsp90 inhibitor radicicol. FEBS J 274:4453–4463. doi:10.1111/j.1742-4658.2007.05974.x 17681020

[9] Marzec M, Eletto D, Argon Y. 2012. GRP94: an HSP90-like protein specialized for protein folding and quality control in the endoplasmic reticulum. Biochim Biophys Acta 1823:774–787. doi:10.1016/j.bbamcr.2011.10.013 22079671 PMC3443595

[10] Munro S, Pelham HRB. 1987. A C-terminal signal prevents secretion of luminal ER proteins. Cell 48:899–907. doi:10.1016/0092-8674(87)90086-9 3545499

[11] Joshi A, Ito T, Picard D, Neckers L. 2022. The mitochondrial HSP90 paralog TRAP1: structural dynamics, interactome, role in metabolic regulation, and inhibitors. Biomolecules 12:880. doi:10.3390/biom12070880 35883436 PMC9312948

[12] Girstmair H, Tippel F, Lopez A, Tych K, Stein F, Haberkant P, Schmid PWN, Helm D, Rief M, Sattler M, Buchner J. 2019. The Hsp90 isoforms from S. cerevisiae differ in structure, function and client range. Nat Commun 10:3626. doi:10.1038/s41467-019-11518-w 31399574 PMC6689086

[13] Pavithra SR, Kumar R, Tatu U. 2007. Systems analysis of chaperone networks in the malarial parasite Plasmodium falciparum. PLoS Comput Biol 3:e168. doi:10.1371/journal.pcbi.0030168 17941702 PMC1976336

[14] Corbett KD, Berger JM. 2010. Structure of the ATP-binding domain of Plasmodium falciparum Hsp90. Proteins 78:2738–2744. doi:10.1002/prot.22799 20635416 PMC2927708

[15] Silva NSM, Torricillas MS, Minari K, Barbosa LRS, Seraphim TV, Borges JC. 2020. Solution structure of Plasmodium falciparum Hsp90 indicates a high flexible dimer. Arch Biochem Biophys 690:108468. doi:10.1016/j.abb.2020.108468 32679196

[16] Stofberg ML, Caillet C, de Villiers M, Zininga T. 2021. Inhibitors of the Plasmodium falciparum Hsp90 towards selective antimalarial drug design: the past, present and future. Cells 10:2849. doi:10.3390/cells10112849 34831072 PMC8616389

[17] Muzenda FL, Stofberg ML, Mthembu W, Achilonu I, Strauss E, Zininga T. 2025. Characterization and inhibition of the chaperone function of Plasmodium falciparum glucose-regulated protein 94 kDa (Pf Grp94). Proteins 93:957–971. doi:10.1002/prot.26779 39670568 PMC11968560

[18] Braumann F, Klug D, Kehrer J, Song G, Feng J, Springer TA, Frischknecht F. 2023. Conformational change of Plasmodium TRAP is essential for sporozoite migration and transmission. EMBO Rep 24:e57064. doi:10.15252/embr.202357064 37306042 PMC10328070

[19] Brandau S, Dresel A, Clos J. 1995. High constitutive levels of heat-shock proteins in human-pathogenic parasites of the genus Leishmania. Biochem J 310 (Pt 1):225–232. doi:10.1042/bj3100225 7646449 PMC1135877

[20] Jamabo M, Bentley SJ, Macucule-Tinga P, Tembo P, Edkins AL, Boshoff A. 2022. In silico analysis of the HSP90 chaperone system from the African trypanosome, Trypanosoma brucei. Front Mol Biosci 9. doi:10.3389/fmolb.2022.947078 PMC9538636 36213128

[21] Mottram JC, Murphy WJ, Agabian N. 1989. A transcriptional analysis of the Trypanosoma brucei hsp83 gene cluster. Mol Biochem Parasitol 37:115–127. doi:10.1016/0166-6851(89)90108-4 2515434

[22] Pizarro JC, Hills T, Senisterra G, Wernimont AK, Mackenzie C, Norcross NR, Ferguson MAJ, Wyatt PG, Gilbert IH, Hui R. 2013. Exploring the Trypanosoma brucei Hsp83 potential as a target for structure guided drug design. PLoS Negl Trop Dis 7:e2492. doi:10.1371/journal.pntd.0002492 24147171 PMC3798429

[23] Meyer KJ, Shapiro TA. 2021. Cytosolic and mitochondrial Hsp90 in cytokinesis, mitochondrial DNA replication, and drug action in Trypanosoma brucei. Antimicrob Agents Chemother 65:e00632-21. doi:10.1128/AAC.00632-21 34424040 PMC8522745

[24] Krukenberg KA, Street TO, Lavery LA, Agard DA. 2011. Conformational dynamics of the molecular chaperone Hsp90. Q Rev Biophys 44:229–255. doi:10.1017/S0033583510000314 21414251 PMC5070531

[25] Jahn M, Rehn A, Pelz B, Hellenkamp B, Richter K, Rief M, Buchner J, Hugel T. 2014. The charged linker of the molecular chaperone Hsp90 modulates domain contacts and biological function. Proc Natl Acad Sci USA 111:17881–17886. doi:10.1073/pnas.1414073111 25468961 PMC4273377

[26] Mansfield CR, Quan B, Chirgwin ME, Eduful B, Hughes PF, Neveu G, Sylvester K, Ryan DH, Kafsack BFC, Haystead TAJ, Leahy JW, Fitzgerald MC, Derbyshire ER. 2024. Selective targeting of Plasmodium falciparum Hsp90 disrupts the 26S proteasome. Cell Chem Biol 31:729–742. doi:10.1016/j.chembiol.2024.02.008 38492573 PMC11031320

[27] Tabassum W, Singh P, Suthram N, Bhattacharyya S, Bhattacharyya MK. 2021. Synergistic action between PfHsp90 inhibitor and PfRad51 inhibitor induces elevated DNA damage sensitivity in the malaria parasite. Antimicrob Agents Chemother 65:e00457-21. doi:10.1128/AAC.00457-21 34097485 PMC8370194

[28] Tabassum W, Bhattacharya M, Bakshi S, Bhattacharyya MK. 2022. Heat shock protein 90 regulates the activity of histone deacetylase Sir2 in Plasmodium falciparum. mSphere 7:e00329-22. doi:10.1128/msphere.00329-22 36121150 PMC9599603

[29] Fangaria N, Rani K, Singh P, Dey S, Kumar KA, Bhattacharyya S. 2022. DNA damage-induced nuclear import of HSP90α is promoted by Aha1. Mol Biol Cell 33:ar140. doi:10.1091/mbc.E21-11-0554 36260391 PMC9727810

[30] Elaimy AL, Ahsan A, Marsh K, Pratt WB, Ray D, Lawrence TS, Nyati MK. 2016. ATM is the primary kinase responsible for phosphorylation of Hsp90α after ionizing radiation. Oncotarget 7:82450–82457. doi:10.18632/oncotarget.12557 27738310 PMC5347704

[31] de Oliveira BCD, Shiburah ME, Paiva SC, Vieira MR, Morea EGO, da Silva MS, Alves C de S, Segatto M, Gutierrez-Rodrigues F, Borges JC, Calado RT, Cano MIN. 2021. Possible involvement of Hsp90 in the regulation of telomere length and telomerase activity during the Leishmania amazonensis developmental cycle and population proliferation. Front Cell Dev Biol 9:713415. doi:10.3389/fcell.2021.713415 34778247 PMC8581162

[32] Toogun OA, Zeiger W, Freeman BC. 2007. The p23 molecular chaperone promotes functional telomerase complexes through DNA dissociation. Proc Natl Acad Sci USA 104:5765–5770. doi:10.1073/pnas.0701442104 17389357 PMC1851566

[33] Tabassum W, Bhattacharyya S, Varunan SM, Bhattacharyya MK. 2021. Febrile temperature causes transcriptional downregulation of Plasmodium falciparum Sirtuins through Hsp90-dependent epigenetic modification. Mol Microbiol 115:1025–1038. doi:10.1111/mmi.14692 33538363

[34] Sahasrabudhe P, Rohrberg J, Biebl MM, Rutz DA, Buchner J. 2017. The plasticity of the Hsp90 co-chaperone system. Mol Cell 67:947–961. doi:10.1016/j.molcel.2017.08.004 28890336

[35] Li Z, Hartl FU, Bracher A. 2013. Structure and function of Hip, an attenuator of the Hsp70 chaperone cycle. Nat Struct Mol Biol 20:929–935. doi:10.1038/nsmb.2608 23812373

[36] Zuehlke A, Johnson JL. 2010. Hsp90 and co-chaperones twist the functions of diverse client proteins. Biopolymers 93:211–217. doi:10.1002/bip.21292 19697319 PMC2810645

[37] Schmid AB, Lagleder S, Gräwert MA, Röhl A, Hagn F, Wandinger SK, Cox MB, Demmer O, Richter K, Groll M, Kessler H, Buchner J. 2012. The architecture of functional modules in the Hsp90 co-chaperone Sti1/Hop. EMBO J 31:1506–1517. doi:10.1038/emboj.2011.472 22227520 PMC3321170

[38] Gray PJ, Prince T, Cheng J, Stevenson MA, Calderwood SK. 2008. Targeting the oncogene and kinome chaperone CDC37. Nat Rev Cancer 8:491–495. doi:10.1038/nrc2420 18511936 PMC2779120

[39] Koulov AV, LaPointe P, Lu B, Razvi A, Coppinger J, Dong M-Q, Matteson J, Laister R, Arrowsmith C, Yates JR 3rd, Balch WE. 2010. Biological and structural basis for Aha1 regulation of Hsp90 ATPase activity in maintaining proteostasis in the human disease cystic fibrosis. Mol Biol Cell 21:871–884. doi:10.1091/mbc.e09-12-1017 20089831 PMC2836968

[40] Rani K, Gotmare A, Maier A, Menghal R, Akhtar N, Fangaria N, Buchner J, Bhattacharyya S. 2024. Identification of a chaperone-code responsible for Rad51-mediated genome repair. J Biol Chem 300:107342. doi:10.1016/j.jbc.2024.107342 38705392 PMC11154708

[41] Dean ME, Johnson JL. 2021. Human Hsp90 cochaperones: perspectives on tissue-specific expression and identification of cochaperones with similar in vivo functions. Cell Stress Chaperones 26:3–13. doi:10.1007/s12192-020-01167-0 33037995 PMC7736379

[42] Chua CS, Low H, Goo KS, Sim TS. 2010. Characterization of Plasmodium falciparum co-chaperone p23: its intrinsic chaperone activity and interaction with Hsp90. Cell Mol Life Sci 67:1675–1686. doi:10.1007/s00018-010-0275-0 20140477 PMC11115557

[43] Chua CS, Low H, Lehming N, Sim TS. 2012. Molecular analysis of Plasmodium falciparum co-chaperone Aha1 supports its interaction with and regulation of Hsp90 in the malaria parasite. Int J Biochem Cell Biol 44:233–245. doi:10.1016/j.biocel.2011.10.021 22100910

[44] Dobson S, Kar B, Kumar R, Adams B, Barik S. 2001. A novel tetratricopeptide repeat (TPR) containing PP5 serine/threonine protein phosphatase in the malaria parasite, Plasmodium falciparum. BMC Microbiol 1:31. doi:10.1186/1471-2180-1-31 11737864 PMC60990

[45] Gitau GW, Mandal P, Blatch GL, Przyborski J, Shonhai A. 2012. Characterisation of the Plasmodium falciparum Hsp70-Hsp90 organising protein (PfHop). Cell Stress Chaperones 17:191–202. doi:10.1007/s12192-011-0299-x 22005844 PMC3273567

[46] Makumire S, Zininga T, Vahokoski J, Kursula I, Shonhai A. 2020. Biophysical analysis of Plasmodium falciparum Hsp70-Hsp90 organising protein (PfHop) reveals a monomer that is characterised by folded segments connected by flexible linkers. PLoS One 15:e0226657. doi:10.1371/journal.pone.0226657 32343703 PMC7188212

[47] Marín-Menéndez A, Monaghan P, Bell A. 2012. A family of cyclophilin-like molecular chaperones in Plasmodium falciparum. Mol Biochem Parasitol 184:44–47. doi:10.1016/j.molbiopara.2012.04.006 22546550

[48] Silva NSM, Seraphim TV, Minari K, Barbosa LRS, Borges JC. 2018. Comparative studies of the low-resolution structure of two p23 co-chaperones for Hsp90 identified in Plasmodium falciparum genome. Int J Biol Macromol 108:193–204. doi:10.1016/j.ijbiomac.2017.11.161 29191421

[49] Wiser MF. 2003. A Plasmodium homologue of cochaperone p23 and its differential expression during the replicative cycle of the malaria parasite. Parasitol Res 90:166–170. doi:10.1007/s00436-003-0835-4 12756555

[50] Chen S, Smith DF. 1998. Hop as an adaptor in the heat shock protein 70 (Hsp70) and hsp90 chaperone machinery. J Biol Chem 273:35194–35200. doi:10.1074/jbc.273.52.35194 9857057

[51] Scheufler C, Brinker A, Bourenkov G, Pegoraro S, Moroder L, Bartunik H, Hartl FU, Moarefi I. 2000. Structure of TPR domain-peptide complexes: critical elements in the assembly of the Hsp70-Hsp90 multichaperone machine. Cell 101:199–210. doi:10.1016/S0092-8674(00)80830-2 10786835

[52] LaCount DJ, Vignali M, Chettier R, Phansalkar A, Bell R, Hesselberth JR, Schoenfeld LW, Ota I, Sahasrabudhe S, Kurschner C, Fields S, Hughes RE. 2005. A protein interaction network of the malaria parasite Plasmodium falciparum. Nature 438:103–107. doi:10.1038/nature04104 16267556

[53] Batista FAH, Seraphim TV, Santos CA, Gonzaga MR, Barbosa LRS, Ramos CHI, Borges JC. 2016. Low sequence identity but high structural and functional conservation: the case of Hsp70/Hsp90 organizing protein (Hop/Sti1) of Leishmania braziliensis. Arch Biochem Biophys 600:12–22. doi:10.1016/j.abb.2016.04.008 27103305

[54] Hombach A, Ommen G, Chrobak M, Clos J. 2013. The Hsp90-Sti1 interaction is critical for Leishmania donovani proliferation in both life cycle stages. Cell Microbiol 15:585–600. doi:10.1111/cmi.12057 23107115 PMC3654555

[55] Morales MA, Watanabe R, Dacher M, Chafey P, Osorio y Fortéa J, Scott DA, Beverley SM, Ommen G, Clos J, Hem S, Lenormand P, Rousselle J-C, Namane A, Späth GF. 2010. Phosphoproteome dynamics reveal heat-shock protein complexes specific to the Leishmania donovani infectious stage. Proc Natl Acad Sci USA 107:8381–8386. doi:10.1073/pnas.0914768107 20404152 PMC2889574

[56] Webb JR, Campos-Neto A, Skeiky YAW, Reed SG. 1997. Molecular characterization of the heat-inducible LmSTI1 protein of Leishmania major. Mol Biochem Parasitol 89:179–193. doi:10.1016/S0166-6851(97)00115-1 9364964

[57] Schmidt JC, Soares MJ, Goldenberg S, Pavoni DP, Krieger MA. 2011. Characterization of TcSTI-1, a homologue of stress-induced protein-1, in Trypanosoma cruzi. Mem Inst Oswaldo Cruz 106:70–77. doi:10.1590/s0074-02762011000100012 21340359

[58] Seraphim TV, Alves MM, Silva IM, Gomes FER, Silva KP, Murta SMF, Barbosa LRS, Borges JC. 2013. Low resolution structural studies indicate that the activator of Hsp90 ATPase 1 (Aha1) of Leishmania braziliensis has an elongated shape which allows its interaction with both N- and M-domains of Hsp90. PLoS One 8:e66822. doi:10.1371/journal.pone.0066822 23826147 PMC3691308

[59] Bartsch K, Hombach-Barrigah A, Clos J. 2017. Hsp90 inhibitors radicicol and geldanamycin have opposing effects on Leishmania Aha1-dependent proliferation. Cell Stress Chaperones 22:729–742. doi:10.1007/s12192-017-0800-2 28455612 PMC5573691

[60] Gunasekera K, Wüthrich D, Braga-Lagache S, Heller M, Ochsenreiter T. 2012. Proteome remodelling during development from blood to insect-form Trypanosoma brucei quantified by SILAC and mass spectrometry. BMC Genomics 13:556. doi:10.1186/1471-2164-13-556 23067041 PMC3545838

[61] Urbaniak MD, Guther MLS, Ferguson MAJ. 2012. Comparative SILAC proteomic analysis of Trypanosoma brucei bloodstream and procyclic lifecycle stages. PLoS One 7:e36619. doi:10.1371/journal.pone.0036619 22574199 PMC3344917

[62] Butter F, Bucerius F, Michel M, Cicova Z, Mann M, Janzen CJ. 2013. Comparative proteomics of two life cycle stages of stable isotope-labeled Trypanosoma brucei reveals novel components of the parasite’s host adaptation machinery. Mol Cell Proteomics 12:172–179. doi:10.1074/mcp.M112.019224 23090971 PMC3536898

[63] Alsford S, Turner DJ, Obado SO, Sanchez-Flores A, Glover L, Berriman M, Hertz-Fowler C, Horn D. 2011. High-throughput phenotyping using parallel sequencing of RNA interference targets in the African trypanosome. Genome Res 21:915–924. doi:10.1101/gr.115089.110 21363968 PMC3106324

[64] Wiser MF, Plitt B. 1987. Plasmodium berghei, P. chabaudi, and P. falciparum: similarities in phosphoproteins and protein kinase activities and their stage specific expression. Exp Parasitol 64:328–335. doi:10.1016/0014-4894(87)90043-9 3315732

[65] McLaughlin SH, Sobott F, Yao Z, Zhang W, Nielsen PR, Grossmann JG, Laue ED, Robinson CV, Jackson SE. 2006. The co-chaperone p23 arrests the Hsp90 ATPase cycle to trap client proteins. J Mol Biol 356:746–758. doi:10.1016/j.jmb.2005.11.085 16403413

[66] Batista FAH, Almeida GS, Seraphim TV, Silva KP, Murta SMF, Barbosa LRS, Borges JC. 2015. Identification of two p23 co-chaperone isoforms in Leishmania braziliensis exhibiting similar structures and Hsp90 interaction properties despite divergent stabilities. FEBS J 282:388–406. doi:10.1111/febs.13141 25369258

[67] Duina AA, Marsh JA, Gaber RF. 1996. Identification of two CyP-40-like cyclophilins in Saccharomyces cerevisiae, one of which is required for normal growth. Yeast 12:943–952. doi:10.1002/(sici)1097-0061(199608)12:10<943::aid-yea997>3.0.co;2-3 8873448

[68] Ratajczak T, Cluning C, Ward BK. 2015. Steroid receptor-associated immunophilins: a gateway to steroid signalling. Clin Biochem Rev 36:31–52. 26224894 PMC4504154

[69] Bernadotte A, Kumar R, Winblad B, Pavlov PF. 2018. In silico identification and biochemical characterization of the human dicarboxylate clamp TPR protein interaction network. FEBS Open Bio 8:1830–1843. doi:10.1002/2211-5463.12521 PMC6212638 30410862

[70] Wu Y, Craig A. 2006. Comparative proteomic analysis of metabolically labelled proteins from Plasmodium falciparum isolates with different adhesion properties. Malar J 5:67. doi:10.1186/1475-2875-5-67 16887017 PMC1559632

[71] Kumar R, Adams B, Musiyenko A, Shulyayeva O, Barik S. 2005. The FK506-binding protein of the malaria parasite, Plasmodium falciparum, is a FK506-sensitive chaperone with FK506-independent calcineurin-inhibitory activity. Mol Biochem Parasitol 141:163–173. doi:10.1016/j.molbiopara.2005.02.007 15850699

[72] Alag R, Bharatham N, Dong A, Hills T, Harikishore A, Widjaja AA, Shochat SG, Hui R, Yoon HS. 2009. Crystallographic structure of the tetratricopeptide repeat domain of Plasmodium falciparum FKBP35 and its molecular interaction with Hsp90 C-terminal pentapeptide. Protein Sci 18:2115–2124. doi:10.1002/pro.226 19691130 PMC2786975

[73] Thommen BT, Dziekan JM, Achcar F, Tjia S, Passecker A, Buczak K, Gumpp C, Schmidt A, Rottmann M, Grüring C, Marti M, Bozdech Z, Brancucci NMB. 2023. Genetic validation of PfFKBP35 as an antimalarial drug target. Elife 12:RP86975. doi:10.7554/eLife.86975 37934560 PMC10629825

[74] Atack TC, Raymond DD, Blomquist CA, Pasaje CF, McCarren PR, Moroco J, Befekadu HB, Robinson FP, Pal D, Esherick LY, Ianari A, Niles JC, Sellers WR. 2020. Targeted covalent inhibition of Plasmodium FK506 binding protein 35. ACS Med Chem Lett 11:2131–2138. doi:10.1021/acsmedchemlett.0c00272 33209191 PMC7667655

[75] Harikishore A, Leow ML, Niang M, Rajan S, Pasunooti KK, Preiser PR, Liu X, Yoon HS. 2013. Adamantyl derivative as a potent inhibitor of Plasmodium FK506 binding protein 35. ACS Med Chem Lett 4:1097–1101. doi:10.1021/ml400306r 24900611 PMC4027365

[76] Bharatham N, Chang MW, Yoon HS. 2011. Targeting FK506 binding proteins to fight malarial and bacterial infections: current advances and future perspectives. Curr Med Chem 18:1874–1889. doi:10.2174/092986711795496818 21466465

[77] Dutta M, Delhi P, Sinha KM, Banerjee R, Datta AK. 2001. Lack of abundance of cytoplasmic cyclosporin A-binding protein renders free-living Leishmania donovani resistant to cyclosporin A. J Biol Chem 276:19294–19300. doi:10.1074/jbc.M009379200 11278494

[78] Hoerauf A, Rascher C, Bang R, Pahl A, Solbach W, Brune K, Röllinghoff M, Bang H. 1997. Host-cell cyclophilin is important for the intracellular replication of Leishmania major. Mol Microbiol 24:421–429. doi:10.1046/j.1365-2958.1997.3401716.x 9159527

[79] Leishmaniasis. 2025. Available from: https://www.who.int/news-room/fact-sheets/detail/leishmaniasis. Retrieved 02 Jul 2025.

[80] Chakraborty A, Das I, Datta R, Sen B, Bhattacharyya D, Mandal C, Datta AK. 2002. A single-domain cyclophilin from Leishmania donovani reactivates soluble aggregates of adenosine kinase by isomerase-independent chaperone function. J Biol Chem 277:47451–47460. doi:10.1074/jbc.M204827200 12244046

[81] Yau W-L, Pescher P, MacDonald A, Hem S, Zander D, Retzlaff S, Blisnick T, Rotureau B, Rosenqvist H, Wiese M, Bastin P, Clos J, Späth GF. 2014. The Leishmania donovani chaperone cyclophilin 40 is essential for intracellular infection independent of its stage-specific phosphorylation status. Mol Microbiol 93:80–97. doi:10.1111/mmi.12639 24811325

[82] Yau W-L, Blisnick T, Taly J-F, Helmer-Citterich M, Schiene-Fischer C, Leclercq O, Li J, Schmidt-Arras D, Morales MA, Notredame C, Romo D, Bastin P, Späth GF. 2010. Cyclosporin A treatment of Leishmania donovani reveals stage-specific functions of cyclophilins in parasite proliferation and viability. PLoS Negl Trop Dis 4:e729. doi:10.1371/journal.pntd.0000729 20614016 PMC2894131

[83] Berman HM, Westbrook J, Feng Z, Gilliland G, Bhat TN, Weissig H, Shindyalov IN, Bourne PE. 2000. The protein data bank. Nucleic Acids Res 28:235–242. doi:10.1093/nar/28.1.235 10592235 PMC102472

[84] Aranda-Chan V, Cárdenas-Guerra RE, Otero-Pedraza A, Pacindo-Cabrales EE, Flores-Pucheta CI, Montes-Flores O, Arroyo R, Ortega-López J. 2024. Insights into peptidyl-prolyl cis-trans isomerases from clinically important protozoans: from structure to potential biotechnological applications. Pathogens 13:644. doi:10.3390/pathogens13080644 39204244 PMC11357558

[85] Potenza M, Galat A, Minning TA, Ruiz AM, Duran R, Tarleton RL, Marín M, Fichera LE, Búa J. 2006. Analysis of the Trypanosoma cruzi cyclophilin gene family and identification of cyclosporin A binding proteins. Parasitology 132:867–882. doi:10.1017/S0031182005009558 16700961

[86] Zhao R, Kakihara Y, Gribun A, Huen J, Yang G, Khanna M, Costanzo M, Brost RL, Boone C, Hughes TR, Yip CM, Houry WA. 2008. Molecular chaperone Hsp90 stabilizes Pih1/Nop17 to maintain R2TP complex activity that regulates snoRNA accumulation. J Cell Biol 180:563–578. doi:10.1083/jcb.200709061 18268103 PMC2234237

[87] Zhao R, Davey M, Hsu Y-C, Kaplanek P, Tong A, Parsons AB, Krogan N, Cagney G, Mai D, Greenblatt J, Boone C, Emili A, Houry WA. 2005. Navigating the chaperone network: an integrative map of physical and genetic interactions mediated by the hsp90 chaperone. Cell 120:715–727. doi:10.1016/j.cell.2004.12.024 15766533

[88] Eckert K, Saliou J-M, Monlezun L, Vigouroux A, Atmane N, Caillat C, Quevillon-Chéruel S, Madiona K, Nicaise M, Lazereg S, Van Dorsselaer A, Sanglier-Cianférani S, Meyer P, Moréra S. 2010. The Pih1-Tah1 cochaperone complex inhibits Hsp90 molecular chaperone ATPase activity. J Biol Chem 285:31304–31312. doi:10.1074/jbc.M110.138263 20663878 PMC2951205

[89] Ahmad M, Afrin F, Tuteja R. 2013. Identification of R2TP complex of Leishmania donovani and Plasmodium falciparum using genome wide in-silico analysis. Commun Integr Biol 6:e26005. doi:10.4161/cib.26005 24505500 PMC3913666

[90] Araujo SA, Martins GH, Quel NG, Aragão AZB, Morea EGO, Borges JC, Houry WA, Cano MIN, Ramos CHI. 2021. Purification and characterization of a novel and conserved TPR-domain protein that binds both Hsp90 and Hsp70 and is expressed in all developmental stages of Leishmania major. Biochimie 182:51–60. doi:10.1016/j.biochi.2020.12.017 33421500

[91] Russell LC, Whitt SR, Chen M-S, Chinkers M. 1999. Identification of conserved residues required for the binding of a tetratricopeptide repeat domain to heat shock protein 90. J Biol Chem 274:20060–20063. doi:10.1074/jbc.274.29.20060 10400612

[92] Chen MX, Cohen PTW. 1997. Activation of protein phosphatase 5 by limited proteolysis or the binding of polyunsaturated fatty acids to the TPR domain. FEBS Lett 400:136–140. doi:10.1016/s0014-5793(96)01427-5 9000529

[93] Yang J, Roe SM, Cliff MJ, Williams MA, Ladbury JE, Cohen PTW, Barford D. 2005. Molecular basis for TPR domain-mediated regulation of protein phosphatase 5. EMBO J 24:1–10. doi:10.1038/sj.emboj.7600496 15577939 PMC544909

[94] Wandinger SK, Suhre MH, Wegele H, Buchner J. 2006. The phosphatase Ppt1 is a dedicated regulator of the molecular chaperone Hsp90. EMBO J 25:367–376. doi:10.1038/sj.emboj.7600930 16407978 PMC1383513

[95] Vaughan CK, Mollapour M, Smith JR, Truman A, Hu B, Good VM, Panaretou B, Neckers L, Clarke PA, Workman P, Piper PW, Prodromou C, Pearl LH. 2008. Hsp90-dependent activation of protein kinases is regulated by chaperone-targeted dephosphorylation of Cdc37. Mol Cell 31:886–895. doi:10.1016/j.molcel.2008.07.021 18922470 PMC2568865

[96] Norris-Mullins B, Vacchina P, Morales MA. 2014. Catalytic activity of a novel serine/threonine protein phosphatase PP5 from Leishmania major. Parasite 21:25. doi:10.1051/parasite/2014027 24890370 PMC4042446

[97] Chaudhuri M. 2001. Cloning and characterization of a novel serine/threonine protein phosphatase type 5 from Trypanosoma brucei. Gene 266:1–13. doi:10.1016/s0378-1119(01)00367-5 11290414

[98] Anderson S, Jones C, Saha L, Chaudhuri M. 2006. Functional characterization of the serine/threonine protein phosphatase 5 from Trypanosoma brucei. J Parasitol 92:1152–1161. doi:10.1645/GE-916R1.1 17304789

[99] Jones C, Anderson S, Singha UK, Chaudhuri M. 2008. Protein phosphatase 5 is required for Hsp90 function during proteotoxic stresses in Trypanosoma brucei. Parasitol Res 102:835–844. doi:10.1007/s00436-007-0817-z 18193284

[100] Lee YT, Jacob J, Michowski W, Nowotny M, Kuznicki J, Chazin WJ. 2004. Human Sgt1 binds HSP90 through the CHORD-Sgt1 domain and not the tetratricopeptide repeat domain. J Biol Chem 279:16511–16517. doi:10.1074/jbc.M400215200 14761955

[101] Ommen G, Chrobak M, Clos J. 2010. The co-chaperone SGT of Leishmania donovani is essential for the parasite’s viability. Cell Stress Chaperones 15:443–455. doi:10.1007/s12192-009-0160-7 19953351 PMC3082645

[102] Coto ALS, Seraphim TV, Batista FAH, Dores-Silva PR, Barranco ABF, Teixeira FR, Gava LM, Borges JC. 2018. Structural and functional studies of the Leishmania braziliensis SGT co-chaperone indicate that it shares structural features with HIP and can interact with both Hsp90 and Hsp70 with similar affinities. Int J Biol Macromol 118:693–706. doi:10.1016/j.ijbiomac.2018.06.123 29959008

[103] Subota I, Julkowska D, Vincensini L, Reeg N, Buisson J, Blisnick T, Huet D, Perrot S, Santi-Rocca J, Duchateau M, Hourdel V, Rousselle J-C, Cayet N, Namane A, Chamot-Rooke J, Bastin P. 2014. Proteomic analysis of intact flagella of procyclic Trypanosoma brucei cells identifies novel flagellar proteins with unique sub-localization and dynamics. Mol Cell Proteomics 13:1769–1786. doi:10.1074/mcp.M113.033357 24741115 PMC4083114

[104] Shimogawa MM, Saada EA, Vashisht AA, Barshop WD, Wohlschlegel JA, Hill KL. 2015. Cell surface proteomics provides insight into stage-specific remodeling of the host-parasite interface in Trypanosoma brucei. Mol Cell Proteomics 14:1977–1988. doi:10.1074/mcp.M114.045146 25963835 PMC4587323

[105] Kundrat L, Regan L. 2010. Balance between folding and degradation for Hsp90-dependent client proteins: a key role for CHIP. Biochemistry 49:7428–7438. doi:10.1021/bi100386w 20704274 PMC3141330

[106] Muller P, Ruckova E, Halada P, Coates PJ, Hrstka R, Lane DP, Vojtesek B. 2013. C-terminal phosphorylation of Hsp70 and Hsp90 regulates alternate binding to co-chaperones CHIP and HOP to determine cellular protein folding/degradation balances. Oncogene 32:3101–3110. doi:10.1038/onc.2012.314 22824801

[107] Kumar R, Pavithra SR, Tatu U. 2007. Three-dimensional structure of heat shock protein 90 from Plasmodium falciparum: molecular modelling approach to rational drug design against malaria. J Biosci 32:531–536. doi:10.1007/s12038-007-0052-x 17536172

[108] Caplan AJ, Ma’ayan A, Willis IM. 2007. Multiple kinases and system robustness: a link between Cdc37 and genome integrity. Cell Cycle 6:3145–3147. doi:10.4161/cc.6.24.5147 18075309 PMC3045565

[109] Lamphere L, Fiore F, Xu X, Brizuela L, Keezer S, Sardet C, Draetta GF, Gyuris J. 1997. Interaction between Cdc37 and Cdk4 in human cells. Oncogene 14:1999–2004. doi:10.1038/sj.onc.1201036 9150368

[110] Kimura Y, Rutherford SL, Miyata Y, Yahara I, Freeman BC, Yue L, Morimoto RI, Lindquist S. 1997. Cdc37 is a molecular chaperone with specific functions in signal transduction. Genes Dev 11:1775–1785. doi:10.1101/gad.11.14.1775 9242486

[111] Li T, Jiang HL, Tong YG, Lu JJ. 2018. Targeting the Hsp90-Cdc37-client protein interaction to disrupt Hsp90 chaperone machinery. J Hematol Oncol 11:59. doi:10.1186/s13045-018-0602-8 29699578 PMC5921262

[112] Bandhakavi S, McCann RO, Hanna DE, Glover CVC. 2003. A positive feedback loop between protein kinase CKII and Cdc37 promotes the activity of multiple protein kinases. J Biol Chem 278:2829–2836. doi:10.1074/jbc.M206662200 12435747

[113] Mandal AK, Lee P, Chen JA, Nillegoda N, Heller A, DiStasio S, Oen H, Victor J, Nair DM, Brodsky JL, Caplan AJ. 2007. Cdc37 has distinct roles in protein kinase quality control that protect nascent chains from degradation and promote posttranslational maturation. J Cell Biol 176:319–328. doi:10.1083/jcb.200604106 17242065 PMC1857360

[114] Backe SJ, Sager RA, Woodford MR, Makedon AM, Mollapour M. 2020. Post-translational modifications of Hsp90 and translating the chaperone code. J Biol Chem 295:11099–11117. doi:10.1074/jbc.REV120.011833 32527727 PMC7415980

[115] Pallavi R, Roy N, Nageshan RK, Talukdar P, Pavithra SR, Reddy R, Venketesh S, Kumar R, Gupta AK, Singh RK, Yadav SC, Tatu U. 2010. Heat shock protein 90 as a drug target against protozoan infections: biochemical characterization of HSP90 from Plasmodium falciparum and Trypanosoma evansi and evaluation of its inhibitor as a candidate drug. J Biol Chem 285:37964–37975. doi:10.1074/jbc.M110.155317 20837488 PMC2992230

[116] Banumathy G, Singh V, Pavithra SR, Tatu U. 2003. Heat shock protein 90 function is essential for Plasmodium falciparum growth in human erythrocytes. J Biol Chem 278:18336–18345. doi:10.1074/jbc.M211309200 12584193

[117] Wiesgigl M, Clos J. 2001. Heat shock protein 90 homeostasis controls stage differentiation in Leishmania donovani. Mol Biol Cell 12:3307–3316. doi:10.1091/mbc.12.11.3307 11694568 PMC60256

[118] Graefe SEB, Wiesgigl M, Gaworski I, Macdonald A, Clos J. 2002. Inhibition of HSP90 in Trypanosoma cruzi induces a stress response but no stage differentiation. Eukaryot Cell 1:936–943. doi:10.1128/EC.1.6.936-943.2002 12477794 PMC138760

[119] Cha SJ, Vega-Rodriguez J, Tao D, Kudyba HM, Hanner K, Jacobs-Lorena M. 2024. Plasmodium female gamete surface HSP90 is a key determinant for fertilization. mBio 15:e03142-23. doi:10.1128/mbio.03142-23 38131664 PMC10865824

[120] Sultan AA, Thathy V, Frevert U, Robson KJH, Crisanti A, Nussenzweig V, Nussenzweig RS, Ménard R. 1997. TRAP is necessary for gliding motility and infectivity of Plasmodium sporozoites. Cell 90:511–522. doi:10.1016/S0092-8674(00)80511-5 9267031

[121] Larreta R, Soto M, Alonso C, Requena JM. 2000. Leishmania infantum: gene cloning of the GRP94 homologue, its expression as recombinant protein, and analysis of antigenicity. Exp Parasitol 96:108–115. doi:10.1006/expr.2000.4553 11052869

[122] Turco SJ, Descoteaux A. 1992. The lipophosphoglycan of Leishmania parasites. Annu Rev Microbiol 46:65–94. doi:10.1146/annurev.mi.46.100192.000433 1444269

[123] Daniyan MO, Przyborski JM, Shonhai A. 2019. Partners in mischief: functional networks of heat shock proteins of Plasmodium falciparum and their influence on parasite virulence. Biomolecules 9:295. doi:10.3390/biom9070295 31340488 PMC6681276

