# Supplementary figures and images for: Hsp90 and associates shaping parasite biology
Source: mSphere. 2025 Sep 24;10(10):e00329-25. doi: 10.1128/msphere.00329-25 (PMC12570473; doi:10.1128/msphere.00329-25)

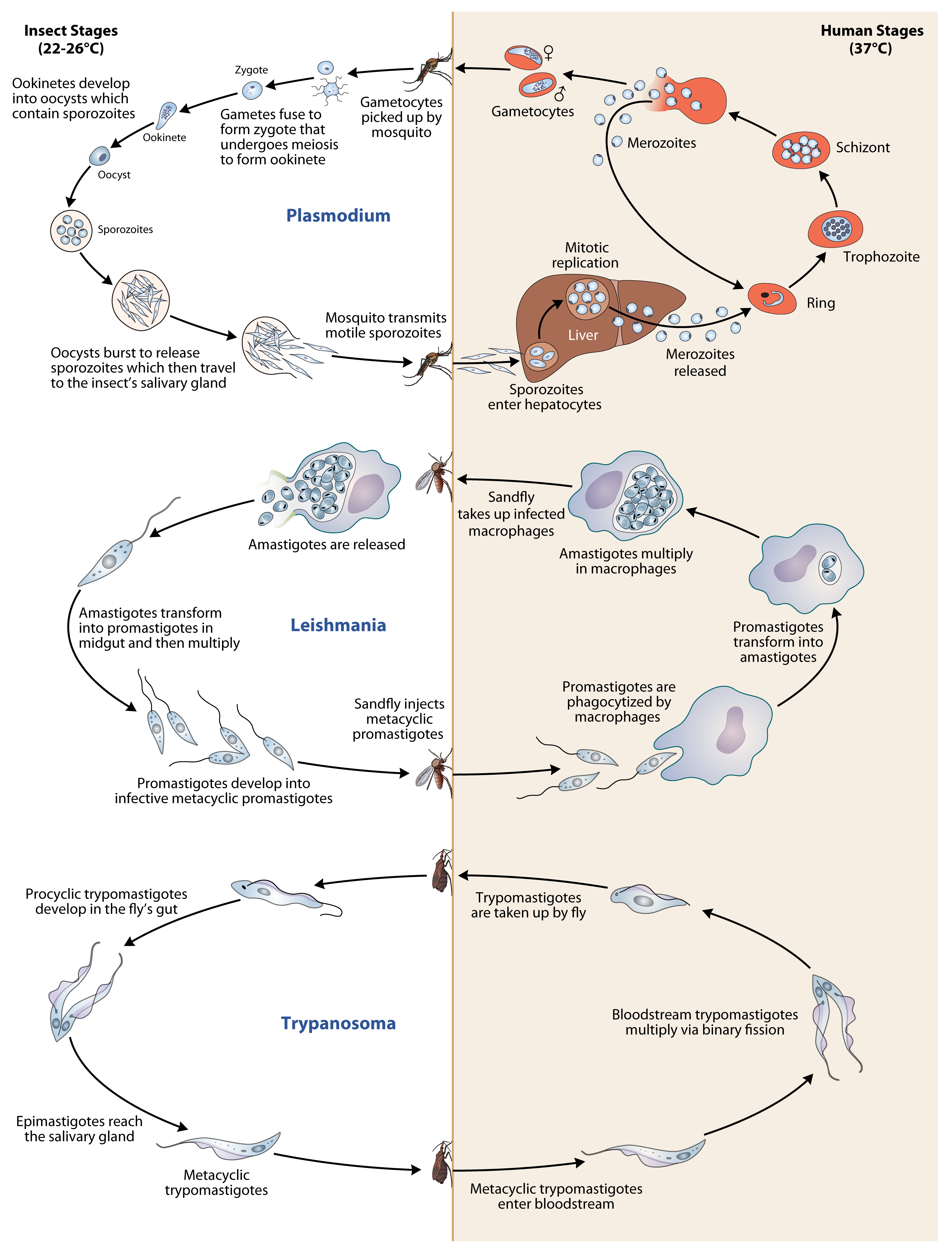

Supplement: Figure S1 — Life cycles of P. falciparum, L. donovani, and T. brucei. [file msphere.00329-25-s0001.tif]

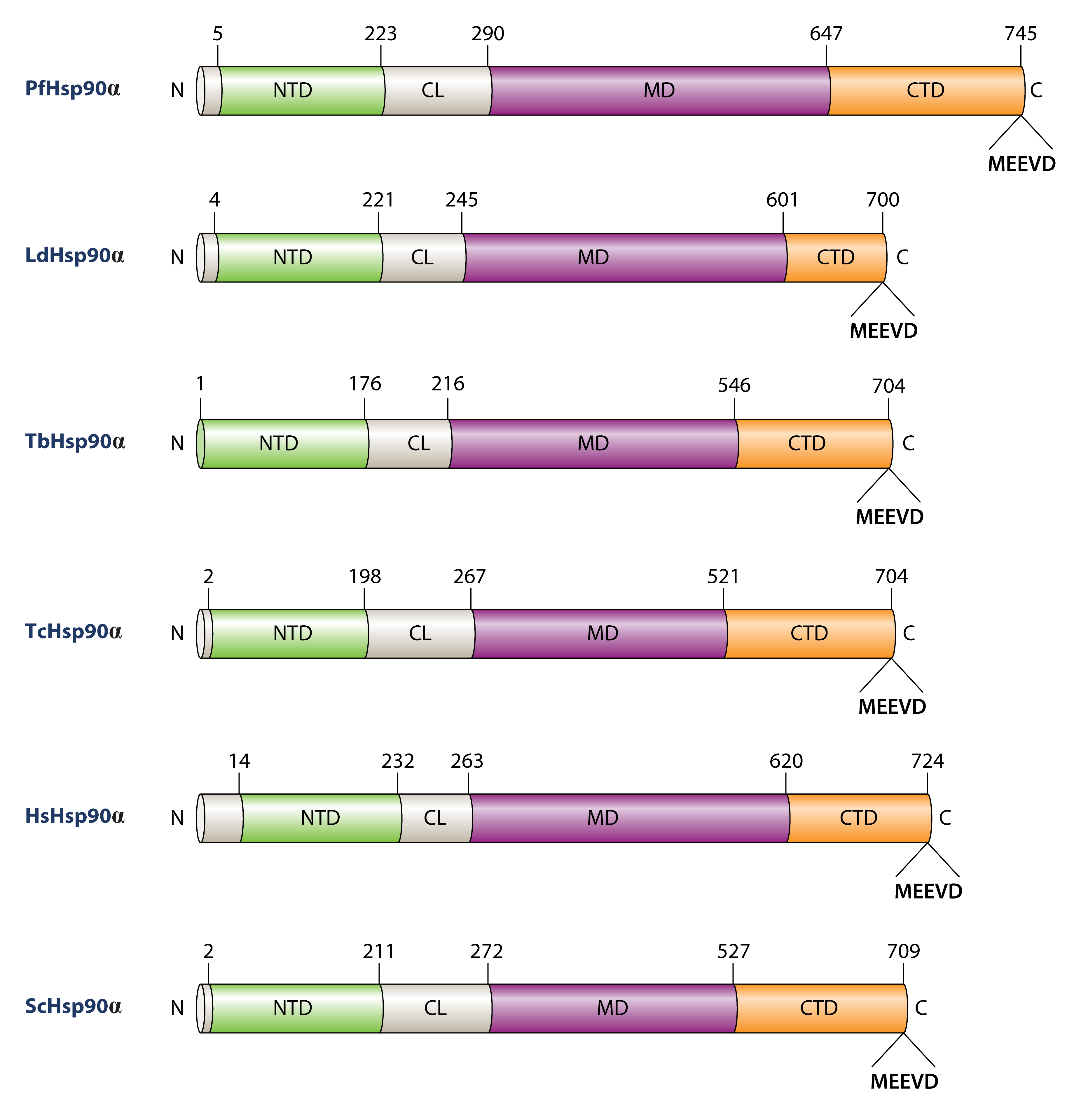

Supplement: Figure S2 — Domain architecture for different Hsp90⍺ orthologs. [file msphere.00329-25-s0002.tif]

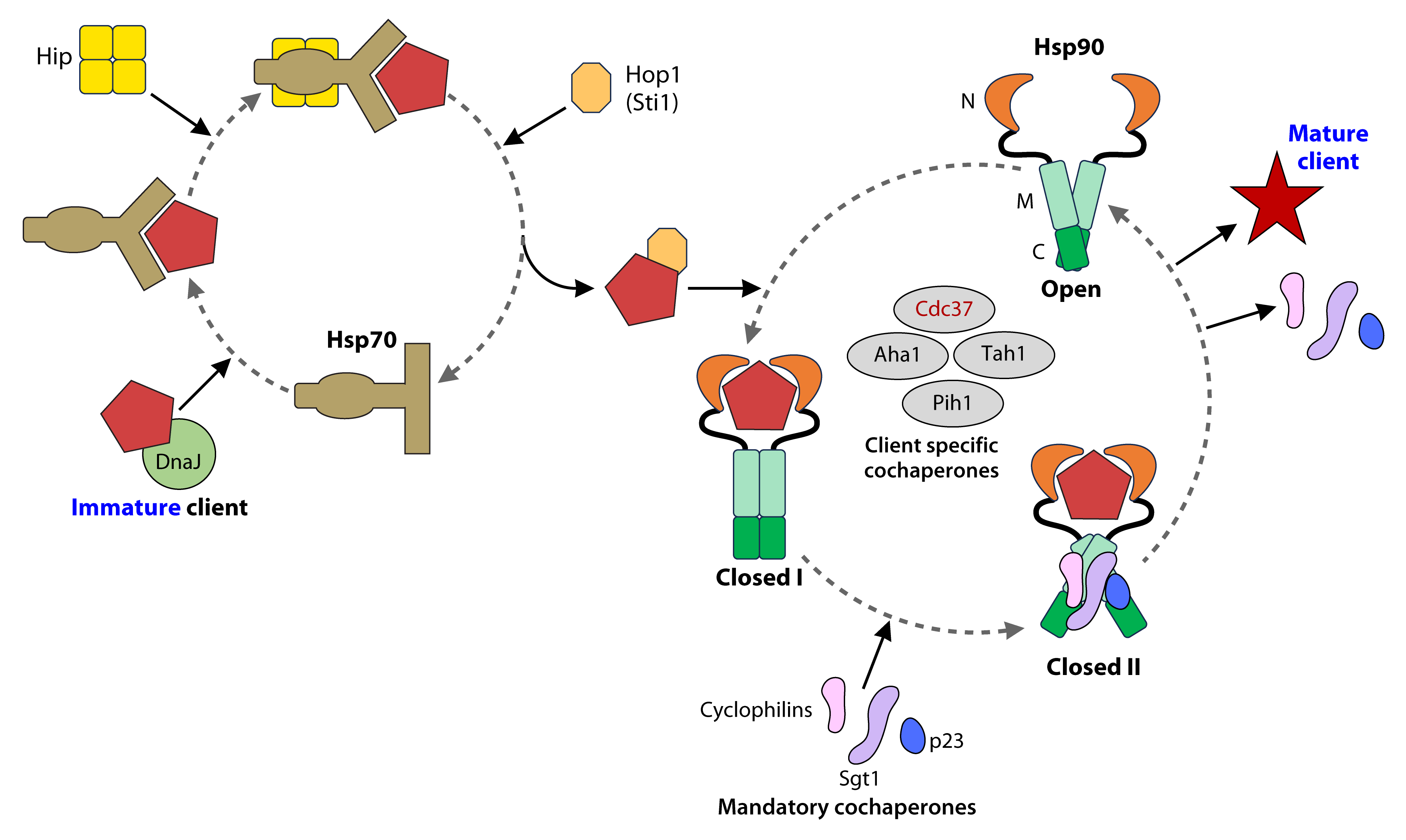

Supplement: Figure S3 — Schematic diagram depicting client maturation through Hsp90 chaperone cycle. [file msphere.00329-25-s0003.tif]
