# Supplementary material for: Hsp90 and associates shaping parasite biology
Source: mSphere. 2025 Sep 24;10(10):e00329-25. doi: 10.1128/msphere.00329-25 (PMC12570473; doi:10.1128/msphere.00329-25)
Supplement: Legends — for Fig. S1 to S3. [file msphere.00329-25-s0004.docx]

**Figure S1: Life cycles of *Plasmodium falciparum*, *Leishmania donovani* and *Trypanosoma brucei*.** Life cycles of these three protozoan parasites shown here depict the shuttling of different stages of these vector-born parasites between the host (at a temperature of 37℃) and their respective vectors (at temperatures ranging between 22 to 26℃).

**Figure S2: Domain architecture for different Hsp90⍺ orthologs.** Residues demarcating each domain in *Plasmodium falciparum* (PfHsp90), *Leishmania donovani* (LdHsp90), *Trypanosoma brucei* (TbHsp90), *Trypanosoma cruzi* (TcHsp90) *Homo sapiens* (HsHsp90) and *Saccharomyces cerevisiae* (ScHsp90) have been mentioned where NTD is N-Terminal Domain (green), CL is Charge Linker domain (white), MD is Middle Domain (purple), and CTD is Charged Linker Domain (orange). Each isoform terminates on the conserved MEEVD motif at the end of the C-terminal domain.

**Figure S3: Schematic diagram depicting client maturation through Hsp90 chaperone cycle.** After synthesis in the ribosome the client proteins are transferred to Hsp70 by DnaJ (J-domain containing Hsp40) proteins. This leads to the conformational change of Hsp70 resulting in tight binding with the client. Hip proteins then stabilize the client bound Hsp70 complex. Hop1 (Sti1 in yeast) facilitates the transfer of the client from Hsp70 to Hsp90 chaperone cycle. The client bound Hsp90 at first exists as closed I form and then upon binding of the mandatory cochaperones: Sgt1, p23 and cyclophilins it is then transformed into the closed II form. At this point one or more auxiliary cochaperones (e.g. Cdc37, Aha1, Tah1, Pih1 etc.) could be involved in a client specific manner. Upon releasing of the mature proteins along with the cochaperones Hsp90 again goes back to the open form and become ready for the next client binding.
