# Supplementary material for: Hsp90 and associates shaping parasite biology
Source: mSphere. 2025 Sep 24;10(10):e00329-25. doi: 10.1128/msphere.00329-25 (PMC12570473; doi:10.1128/msphere.00329-25)
Supplement: Supplemental tables — Tables S1 and S2. [file msphere.00329-25-s0005.docx]

**Table S1: List of Hsp90 Isoforms found in *Plasmodium*, *Leishmania* and *Trypanosoma***

| **Hsp90 Isoform** | **Accession Number/Gene ID** | **Putative/ Characterized** | **Localization** | **Constitutive/Inducible** |
| --- | --- | --- | --- | --- |
| ***Plasmodium falciparum:*** | | | |  |
| PfHsp90/PfHsp86 | PF3D7_0708400 | Experimentally  characterized (1) | Cytoplasm  (Experimentally determined) | Inducible |
| PfGrp94 | PF3D7_1222300 | Experimentally characterized (2) | Endoplasmic Reticulum  (Predicted) | Inducible |
| PfTrap1/PfHsp90_M | PF3D7_1118200 | Experimentally characterized (3) | Mitochondria (Predicted) | Inducible |
| PfHsp90_constitutive | PF3D7_1443900 | Putative | - | Constitutive |
| ***Leishmania major:*** | | | | |
| LmHsp83 | LmjF.33.0312 | Putative | Cytoplasm | Inducible |
| LmGrp94/LmGp96/  Endoplasmin | LmjF.29.0760 | Putative | Endoplasmic Reticulum | **-** |
| LmTrap1/LmHsp75 | LmjF33.2390 | Putative | Mitochondria | **-** |
| ***Leishmania donovani:*** | | | | |
| LdHsp83 | CAJ1992000.1  (GenBank) | Experimentally characterized (4,5) | Cytoplasm | Inducible |
| LdGrp94/LdGp96/  Endoplasmin | CAJ1990547.1  (GenBank) | Experimentally characterized (6) | Endoplasmic Reticulum | **-** |
| LdTrap1/LdHsp75 | U.I. | - | - | **-** |
| ***Trypanosoma brucei:*** | | | | |
| TbHsp83 | Tb927.10.10980 | Experimentally characterized (7,8) | Cytoplasm | Inducible |
| TbGrp94/TbHsp90B1 | Tb927.3.3580 Tbg972.3.3850 | Putative | Endoplasmic Reticulum | **-** |
| TbTrap1/TbHsp84 | Tb427tmp.02.0250 | Experimentally characterized (8) | Mitochondria | **-** |
| ***Trypanosoma cruzi:*** | | | | |
| TcHsp83 | TcCLB.507713.30 | Experimentally  characterized (9,10) | Cytoplasm | Inducible |
| TcGrp94/TcHsp90B1/  TcGp96 | TcCLB.506989.190 | Putative | Endoplasmic Reticulum | **-** |
| TcTrap1/TcHsp84 | TcCLB.504153.310 | Putative | Mitochondria | **-** |

U.A. - Unidentified

**Table S2: List of co-chaperones found in *Plasmodium*, *Leishmania* and *Trypanosoma***

| **Human** | **Yeast** | ***Plasmodium***  ***falciparum*** | ***Leishmania*** | ***Trypanosoma***  ***brucei*** | ***Trypanosoma***  ***cruzi*** |
| --- | --- | --- | --- | --- | --- |
| Hop | Sti1 | PfHop (PF3D7_1434300) | LbSti1 (LbrM.33.0350) | TbSti1 (AF107772.1) | Tc_MARK_9009  C4B63_59g115 |
| Aha1 | Aha1, Hch1 | PfAha1 (PF3D7_0306200) | LbAha1 (XP_001563948.1) | TbAha1  (Tb927.10.13710) | TcCLB.507.993.150  Tc_MARK_4860  C4B63_4g357 |
| p23 | Sba1 | Pfp23A (PF3D7_1453700) Pfp23B (PF3D7_0927000) | Lbp23A (XP_001568545.1)  Lbp23B (XP_001564309.1) | Tbp23A (Tb927.9.10230)  Tbp23B (Tb927.10.2620) | TcCLB.509.551.70  TcCLB.506.407.60  C4B63_2g235  C4B63_47g40 |
| FKBP38 | - | PfFKBP35 (PF3D7_1247400) | LdFK506  LdCyp (AAD46565.1) | TbFKBPL (XP_828079.1)  TbCyp40 (XP_827280.1) | TcCLB.511.353.10  Tc_MARK_4665  C4B63_157g28  C4B63_171g30 |
| Pih1 | Pih1 | PfPih1 (PF3D7_1235000) | LdPih1 (Ldbpk_354400.1) | TbPih1  (Tb927.9.10490) | TcCLB.506.147.150  Tc_MARK_4354  C4B63_80g31 |
| Tah1 | Tah1 | PfRPAP3/PfTah1 (PF3D7_0213500) | LdTah1 (Ldbpk_081020.1) | U.I. | U.I. |
| PP5 | Ppt1 | PfPP5 (PF3D7_1355500) | LmPP5  ([XP_001682421](https://www.ncbi.nlm.nih.gov/protein/XP_001682421)) | TbPP5 (Tb927.10.13670) | TcCLB.507.993.190  C4B63_4g368 |
| Sgt1 | Sgt1 | PfCBP (PF3D7_0933200) | LmSgt  (LmjF.30.2740) | TbSgt  (Tb927.6.4000) | TcCLB.511.737.10  Tc_MARK_2022  C4B63_18g260 |
| CHIP | - | PfCHIP (PF3D7_0527500) | U.I. | U.I. | U.I. |
| Cdc37/P50 | Cdc37 | U.I. | U.I. | U.I. | U.I. |

U.I. – Unidentified

References:

1. Pallavi R, Roy N, Nageshan RK, Talukdar P, Pavithra SR, Reddy R, et al. Heat shock protein 90 as a drug target against protozoan infections: Biochemical characterization of HSP90 from plasmodium falciparum and Trypanosoma evansi and evaluation of its inhibitor as a candidate drug. Journal of Biological Chemistry. 2010;285(49).

2. Murillo-Solano C, Dong C, Sanchez CG, Pizarro JC. Identification and characterization of the antiplasmodial activity of Hsp90 inhibitors. Malar J. 2017;16(1).

3. Sultan AA, Thathy V, Frevert U, Robson KJH, Crisanti A, Nussenzweig V, et al. TRAP is necessary for gliding motility and infectivity of Plasmodium sporozoites. Cell [Internet]. 1997 Aug 8 [cited 2025 Aug 5];90(3):511–22. Available from: https://pubmed.ncbi.nlm.nih.gov/9267031/

4. Vergnes B, Gourbal B, Girard I, Sundar S, Drummelsmith J, Ouellette M. A Proteomics Screen Implicates HSP83 and a Small Kinetoplastid Calpain-related Protein in Drug Resistance in Leishmania donovani Clinical Field Isolates by Modulating Drug-induced Programmed Cell Death. Molecular & Cellular Proteomics [Internet]. 2007 Jan 1 [cited 2025 Jun 30];6(1):88–101. Available from: https://www.sciencedirect.com/science/article/pii/S153594762031433X

5. Mansuri R, Diwan A, Kumar Mundotiya P, Singh J. Structural Analysis and In-Silico Inhibitor Interaction Studies of Leishmania donovani Heat-Shocked Proteins 83 (HSP83). Int J Life Sci Pharma Res. 2022 Dec 24;P8–18.

6. Descoteaux A, Avila HA, Zhang K, Turco SJ, Beverley SM. Leishmania LPG3 encodes a GRP94 homolog required for phosphoglycan synthesis implicated in parasite virulence but not viability. EMBO J [Internet]. 2002 Sep 2 [cited 2025 Jun 30];21(17):4458–69. Available from: /doi/pdf/10.1093/emboj/cdf447?download=true

7. Pizarro JC, Hills T, Senisterra G, Wernimont AK, Mackenzie C, Norcross NR, et al. Exploring the Trypanosoma brucei Hsp83 Potential as a Target for Structure Guided Drug Design. PLoS Negl Trop Dis. 2013;7(10).

8. Meyer KJ, Shapiro TA. Cytosolic and mitochondrial Hsp90 in cytokinesis, mitochondrial DNA replication, and drug action in trypanosoma brucei. Antimicrob Agents Chemother. 2021;65(11).

9. Palmer G, Louvion JF, Tibbetts RS, Engman DM, Picard D. Trypanosoma cruzi heat-shock protein 90 can functionally complement yeast. Mol Biochem Parasitol [Internet]. 1995 Mar 1 [cited 2025 Jun 30];70(1–2):199–202. Available from: https://www.sciencedirect.com/science/article/pii/016668519500007N

10. Dragon EA, Sias SR, Kato EA, Gabe JD. The Genome of Trypanosoma cruzi Contains a Constitutively Expressed, Tandemly Arranged Multicopy Gene Homologous to a Major Heat Shock Protein. Mol Cell Biol [Internet]. 1987 Mar 1 [cited 2025 Jun 30];7(3):1271–5. Available from: https://www.tandfonline.com/doi/abs/10.1128/mcb.7.3.1271-1275.1987
